# Metabolic syndrome severity and the energetic cost of brain network transitions: A normative modeling study of accelerated brain aging

**DOI:** 10.1162/IMAG.a.1282

**Published:** 2026-06-22

**Authors:** Hyun Soo Park, Bumseok Jeong

**Affiliations:** Graduate School of Medical Science and Engineering, Korea Advanced Institute of Science and Technology, Yuseong-gu, Daejeon, Republic of Korea

**Keywords:** metabolic syndrome, network control theory, normative modeling, structural connectome, cognitive function

## Abstract

Although metabolic syndrome is a modifiable risk factor for functional decline in various organs, the brain network mechanisms linking metabolic cost to functional vulnerability remain poorly understood. We applied network control theory to diffusion magnetic resonance imaging-derived connectomes in 25,697 adults aged 40–70 years to quantify the energetic cost of engaging 8 large-scale brain networks. Metabolic syndrome was defined according to established criteria, with a composite score (0–5) based on the presence of central obesity, elevated triglycerides, reduced high-density lipoprotein cholesterol, hypertension, and impaired glycemic control. Normative modeling provided age-adjusted network deviations and brain age gap estimates. Associations with metabolic indicators and behavioral cognitive performance (7 tasks in UK Biobank) were tested, and meta-analytic mapping (123 cognitive states in Neurosynth) was used to identify at-risk domains. Severity of metabolic syndrome was associated with distinct, network-specific energetic alterations, including progressively increased subcortical control energy cost, decreased visual, and threshold changes in attentional and executive networks. The control energy profiles of the subcortical and visual networks exhibited accelerated brain age gap trajectories, reflecting significant departures from normative age-matched expectations. Waist circumference was the strongest metabolic predictor, with effects amplified in older age. Partial least squares correlation analysis revealed associations of metabolic profile (central obesity and dyslipidemia) with higher subcortical/dorsal attention/control and lower visual network energy. Vulnerability mapping indicated that episodic memory, spatial navigation, and processing speed were the domains at most at risk in relation to higher metabolic syndrome severity scores. Behavioral testing confirmed lower memory and processing speed scores in participants with metabolic syndrome. Overall, metabolic syndrome reshapes the brain’s energetic landscape, accelerates functional brain aging, and selectively impairs memory-related networks. Network control theory-derived metrics offer a mechanistic and sensitive biomarker linking metabolic health to cognitive function. Central adiposity was the primary modifiable driver, underscoring the importance of early, targeted metabolic interventions to preserve network efficiency and cognitive resilience.

## 1 Introduction

Metabolic syndrome (MetS), defined by central obesity, hypertension, hyperglycemia, and dyslipidemia, affects approximately one-quarter of adults worldwide (Alberti et al., 2009; Lemieux & Despres, 2020; Rochlani et al., 2017; Zhang et al., 2024) and increases the risks of cardiovascular, cerebrovascular, and neurodegenerative disease (Qureshi, Collister, et al., 2024; Qureshi, Topiwala, et al., 2024; Zouridis et al., 2025). Large-scale population studies using the UK Biobank (UKB) have recently confirmed that MetS is associated with widespread reductions in gray matter volume, hippocampal atrophy, and a higher burden of white matter hyperintensities (Du et al., 2024; Kang et al., 2023; Kolbeinsson et al., 2020; Qureshi, Collister, et al., 2024; Qureshi, Topiwala, et al., 2024; Zuo et al., 2024). Beyond systemic effects, MetS is consistently associated with cognitive decline, particularly in executive function, processing speed, and working memory (Koutsonida et al., 2022; Yates et al., 2012). While these domains depend on efficient communication across large-scale brain networks, most studies have focused on structural alterations, offering limited insight into the dynamic, system-level processes underlying cognitive vulnerability (Dove et al., 2024; Jensen et al., 2025; Qureshi, Topiwala, et al., 2024).

Network control theory (NCT) bridges this gap by modeling neural activity propagation through an individual’s structural connectome, quantifying the control energy required to transition between brain states (Parkes et al., 2024; Wu et al., 2024; Zoller et al., 2021). This systems-engineering approach offers mechanistic insight into how MetS-related white matter disruptions, driven by chronic inflammation, endothelial dysfunction, and insulin resistance (Cai & Liu, 2012; Rashid et al., 2021; Rochlani et al., 2017; Yates et al., 2012), increase the energetic cost of engaging brain networks. We specifically utilize diffusion weighted imaging (DWI)-derived structural connectivity rather than functional connectivity, because the structural connectome provides the physical scaffold that constrains neural signaling. In the context of MetS, using structural connectome allows us to mechanistically quantify how physical damage to the brain’s communication relays directly increases the energetic cost of achieving functional states. Unlike conventional graph analysis, which describes static properties (e.g., connectivity strength), NCT directly measures the energetic demands of state transition, thereby mapping structural damage onto functional cost (Gu et al., 2015; Medaglia et al., 2017). This approach is uniquely suited to MetS, as it leverages personalized structural connectomes to quantify individual differences in predicted control energy costs. By doing so, it maps specific network vulnerabilities that may vary significantly among individuals, effectively translating heterogeneous structural damage into a standardized metric of functional cost. By quantifying not only whether a network is altered but also how much additional energy is required for neural state transitions (the dynamic movement between brain activation patterns), NCT provides a direct systems-level link between structural damage and cognitive performance. By calculating these energy costs for individuals across the spectrum of cardiometabolic health, we can map how the energetic demands of brain function scale with increasing MetS severity.

Control energy, as defined in NCT, represents the minimum energetic cost to achieve a desired brain state transition and changes systematically with age, lifestyle, and health status (Gu et al., 2015; He et al., 2022; Stanford et al., 2024). It is important to clarify that this metric is a model-based quantity reflecting the theoretical difficulty or cost of neural state transitions given a specific white-matter architecture. Unlike PET-based measures of glucose metabolism or fMRI-based oxygen utilization, NCT-derived energy serves as a mathematical proxy for network-level efficiency. By modeling the brain’s structural connectome as a linear dynamical system, we can evaluate how MetS reshapes the energetic landscape of the brain, identifying which networks become harder to engage as metabolic symptoms worsen.

Traditional group-level comparisons dilute individual deviations from normative trajectories (Rutherford et al., 2022). Normative modeling overcomes this limitation by estimating a probability distribution as a covariate function, such as age and sex, from large population-based datasets, and then expresses each measurement as a percentile (z-score) within that distribution (Lawn et al., 2024; Rutherford et al., 2022). This approach preserves nonlinear age effects, detects subtle departures from typical aging within age-matched peers, and identifies outliers at the single-participant level with prognostic and intervention-relevant implications (Rutherford et al., 2023). NCT-related metrics show a systematic decline and subsequent readjustments with age, a pattern thought to reflect reduced neural growth and plasticity (Sun et al., 2023; Tang et al., 2017). Thus, applying normative modeling to NCT-derived control energy can identify networks with abnormally high or low energetic demands relative to normal aging, linking these deviations to cardiometabolic cost and cognitive decline.

**Fig. 1.**
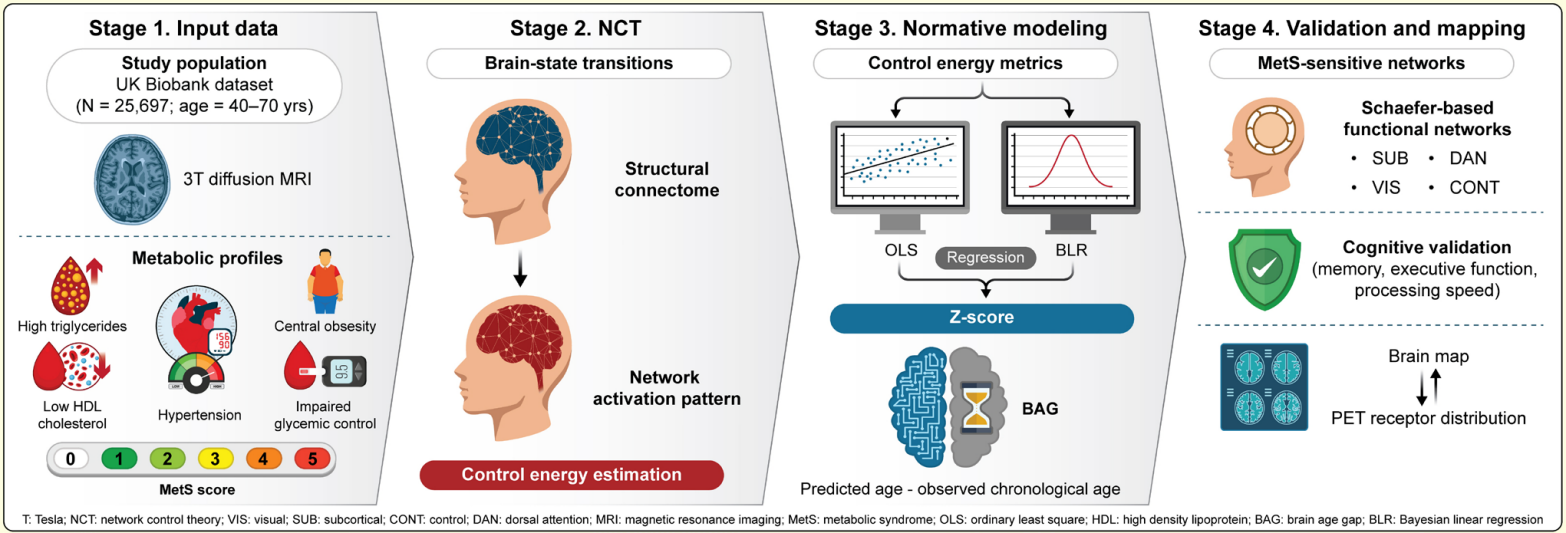
Schematic overview of the study workflow. The analysis consists of four main stages. Stage 1: Data acquisition from the UK Biobank (N = 25,697), including 3T diffusion MRI and metabolic profiling based on five established criteria. Stage 2: Implementation of Network Control Theory (NCT) to estimate the control energy required for brain state activation of eight canonical networks. Stage 3: Normative modeling using Bayesian Linear Regression (BLR) to drive age-adjusted z-scores and brain age gaps (BAG) for each energy metric. Stage 4: Validation of MetS-sensitive networks through cognitive performance testing, meta-analytic cognitive mapping, and spatial colocalization with PET and cell type-specific gene expression maps.

We hypothesized that MetS disrupts structural connectivity to change the control energy required for transitions into specific cognitive networks, and that these network-level energy alterations contribute to observed cognitive impairments. Leveraging multimodal neuroimaging, detailed metabolic profiling, and cognitive assessments from the UKB cohort, we aimed to (i) characterize how MetS severity and individual cardiometabolic components influence the control energy of large-scale brain networks and (ii) link these alterations to observed cognitive performance. Specifically, we calculated the vulnerability to reach meta-analytic functional maps (derived from Neurosynth) and correlated these energetic costs with the individual cognitive assessment scores available within the UKB cohort. The overall analysis framework of this study is summarized in Figure 1. By integrating network neuroscience with metabolic profiling, this study sought to identify control energy-based biomarkers reflecting both metabolic and cognitive resilience, with potential applications in precision prevention and targeted intervention.

## 2 Methods

### 2.1 Study participants

**Fig. 2.**
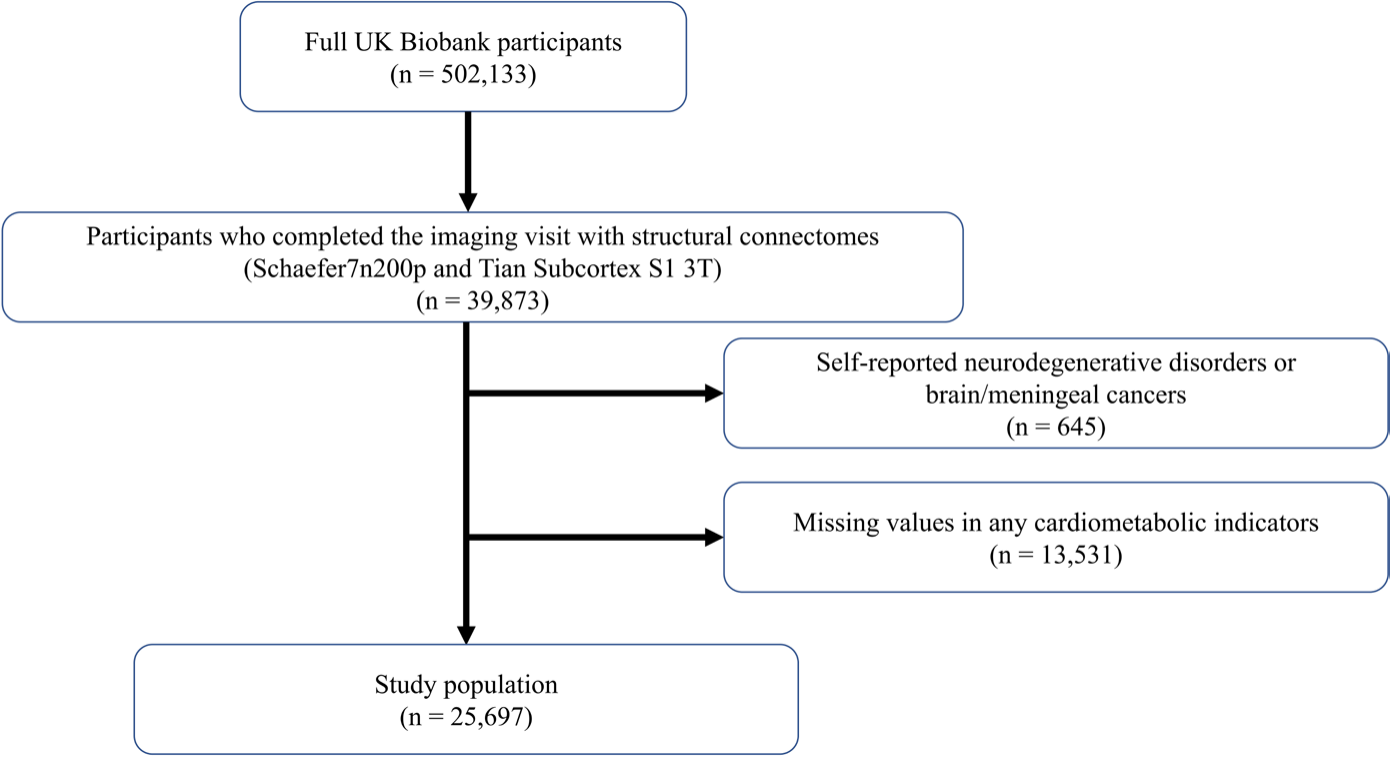
Study flowchart.

Data were drawn from the UKB, a prospective cohort of >500,000 adults aged 40–69 years at baseline (Sudlow et al., 2015). The UKB was approved by the North West Multicenter Research Ethics Committee (11/NW/0382), and all participants provided written informed consent. From the full UKB dataset of 502,133 participants, we first identified participants who had completed the imaging visit (instance 2, conducted after 2014) with non-missing 3T diffusion MRI-derived structural connectomes generated using hybrid parcellation combining the Schaefer 200-region cortical atlas (7 networks) with the Melbourne Subcortex Atlas (Scale 1), yielding 216 nodes (*n* = 39,873) (Mansour et al., 2023; Schaefer et al., 2018; Tian et al., 2020). Structural connectivity was processed using the publicly available pipeline (Mansour et al., 2023), which applies standardized multimodal, multiscale connectome construction. Next, we excluded participants who self-reported neurodegenerative disorders or brain/meningeal cancers (*n* = 645), as documented in Supplementary Table S1. Finally, we removed participants with missing values in any cardiometabolic indicators used to define MetS criteria (*n* = 13,531) (Alberti et al., 2009). The final analytic sample comprised 25,697 participants (Fig. 2).

### 2.2 Cardiometabolic and cognitive assessment

Cardiometabolic indicators included waist circumference, high-density lipoprotein cholesterol (HDL), triglycerides, systolic blood pressure (SBP), diastolic blood pressure (DBP), and glycated hemoglobin (HbA1c). SBP and DBP were measured twice, and their values averaged. MetS was defined according to the 2009 Joint Interim Statement (Alberti et al., 2009), as ≥3 of the follow five criteria: (1) increased waist circumference (men ≥ 102 cm; women ≥ 88 cm), (2) elevated triglycerides (≥1.7 mmol/L or 150 mg/dL), (3) reduced HDL (<1.03 mmol/L or 40 mg/dL for men; <1.29 mmol/L or 50 mg/dL for women), (4) elevated blood pressure (SBP ≥ 130 mmHg and/or DBP ≥ 85 mmHg), and (5) impaired glycemic control (HbA1c ≥ 39 mmol/mol or 5.7%). HbA1c was used as a proxy for fasting glucose, which was unavailable in the dataset (American Diabetes Association, 2010; Qureshi, Topiwala, et al., 2024). A MetS score (range 0–5) was assigned based on the number of criteria met.

Cognitive performance was assessed using seven UKB tasks: fluid intelligence (FI), matrix pattern completion (MPC), paired associate learning (PAL), symbol digit substitution (SDS), trail making test A (TMT-A), trail making test B (TMT-B), and backward digit span (BDS). These tasks evaluated reasoning, executive function, working memory, and processing speed (Fawns-Ritchie & Deary, 2020). Raw data preprocessing involved recoding non-numeric responses (e.g., “trail not completed”, “abandoned”) as missing values. Missing cognitive data were imputed using multivariate imputation by chained equations with 10 iterations and a fixed random seed, preserving multivariate relationships under the missing-at-random assumption (Azur et al., 2011). Relevant UKB field numbers are given in Supplementary Table S2.

### 2.3 NCT analysis

#### 2.3.1 NCT implementation

NCT was applied to estimate the energetic cost of transitioning between brain states using individual structural connectomes derived from diffusion MRI. The approach models the brain as a continuous-time, linear time-invariant (LTI) system and computes the optimal control energy required to steer the system from an initial state ${\text{x}}_{0}$ to a target state $x_{f}$ within a fixed time horizon T.

Before applying NCT, we normalized each structural connectome to ensure system stability:

Let A denote the structural connectivity matrix and $\lambda_{max\;}$ its largest eigenvalue magnitude:

$$\lambda_{max}=\max\left(\left|\lambda_{i}\right|\right),\;\forall \;\lambda_{i}\in \;eig\left(A\right).$$

The normalized connectome was computed as Karrer et al. (2020):

$$A_{norm}=\;\frac{A}{c\;+\;\lambda_{max}}-I,$$

where c is a constant, set to 1 in this study, and Iis the identity matrix.

To estimate the energy required to steer brain dynamics between cognitive states, we modeled brain dynamics as

$$\frac{dx\left(t\right)}{dt}=\;A_{norm}x\left(t\right)+\;Bu\left(t\right),$$

where B is the control input matrix (identity matrix, assuming full control over all nodes), x(t)is the state vector representing the activity level of brain regions at time *t*, and u(t) is the external control input applied to the system. Given an initial state $x_{0}$ and a target state $x_{f}$, our goal was to determine the optimal control input u(t) and corresponding state trajectory x(t) that minimize the following quadratic cost function:

$$J\;=\;\int_{0}^{T}\left[{\left|\left|u\left(t\right)\right|\right|}^{2}+\;\rho {\left|\left|S\left(x\left(t\right)-\;x_{r}\right)\right|\right|}^{2}\right]dt,\;$$

where ρ > 0 is a regularization parameter that governs the trade-off between control energy and trajectory deviation, *S* is a diagonal weighting matrix specifying which brain regions to penalize, and $x_{r}$ is the reference state (zero vector by default). When S=I and $x_{r}=0$ , the cost reflects deviation from zero neural activity across all regions.

The optimal control solution was derived by constructing a joint state-costate system based on the Hamiltonian formulation:

$$\frac{d}{dt}\left[\begin{array}{c} x\left(t\right)\\ \lambda \left(t\right) \end{array}\right]=\;M\;\left[\begin{array}{c} x\left(t\right)\\ \lambda \left(t\right) \end{array}\right]+\;c,$$

where λ(t) is the costate vector, and the joint system matrix M and constant vector c are given by

$$M\;=\;\left[\begin{array}{cc} A_{norm} & -\left(\frac{1}{2\rho }\right)BB^{T}\\ -2S & -A_{norm}^{T} \end{array}\right],\;c=\left[\begin{array}{c} 0\\ 2Sx_{r} \end{array}\right].$$

The solution to the system at time T can be expressed analytically as

$$\left[\begin{array}{c} x\left(T\right)\\ \lambda \left(T\right) \end{array}\right]=e^{MT}\left[\begin{array}{c} x_{0}\\ \lambda_{0} \end{array}\right]+\left(e^{MT}-I\right){\text{M}}^{-1}c.$$

We first computed the matrix exponential $e^{MT}$ and partitioned it into four blocks:

$$e^{MT}=\;\left[\begin{array}{c} E_{11}\;\;\;E_{12}\\ E_{21}\;\;\;E_{22} \end{array}\right].$$

The initial costate $\lambda_{0}$ was solved via

$$\lambda_{0}=E_{12}^{-1}\left(x_{f}-E_{11}x_{0}-\left[\left(E_{11}-I\right)E_{12}\right]c\right).$$

To obtain the full state trajectory, we numerically simulated the joint system dynamics over the interval [0, T] using a discretized time step (Δt = 0.001 T). At each time step, the system was updated via

$$z\left(t+\;\Delta t\right)=A_{d}z\left(t\right)+B_{d},$$

where z(t)=[x(t);λ(t)], $A_{d}=e^{M\Delta t}$ , and $B_{d}=\left(A_{d}-I\right)c$ . After simulating the system, the optimal control inputs were computed as

$$u\left(t\right)=\;-\left(\frac{1}{2\rho }\right)B^{\text{T}}\;\lambda \left(t\right).$$

Finally, total control energy was calculated as

$$E\;=\;\int_{0}^{T}{\left|\left|u\left(t\right)\right|\right|}^{2}dt\;$$

using Simpson’s rule for numerical integration. The analysis was implemented in Python using the nctpy package, an open-source toolkit for simulating and analyzing network control in brain systems (Parkes et al., 2024).

#### 2.3.2 Definition of activation energy

Activation energy was defined as the optimal control energy required to activate each canonical brain network from a zero-baseline state (He et al., 2022). The initial state $x_{0}$ was set to a zero vector (no activity across all regions), while the target state $x_{f}$ represented the activation pattern of one network. Activation energy was calculated for eight canonical brain networks—visual (VIS), somatomotor (SMN), dorsal attention (DAN), salience/ventral attention (VAN), limbic (LIM), control (CONT), default mode (DMN), and subcortical (SUB)—by setting the initial state to a mean-centered baseline and the target state to the activation pattern of a given network. This allowed us to estimate the relative energetic cost of engaging each network under identical model constraints.

For each of the eight functional brain networks, the corresponding state vector was constructed by assigning a value of 1 to nodes belonging to the network and 0 elsewhere. Control energy was then computed using the framework described above, with

$$x_{0}=0,x_{f}=\text{network activation pattern}.$$

The resulting activation energy for each network was calculated by integrating the squared control inputs over time. Importantly, setting the initial state to zero does not imply that the brain is entirely inactive at baseline. Rather, it serves as a mean-centred reference, allowing for relative comparisons across network transitions. This allows the control energy metric to serve as a standardized measure of the structural capacity of the individual’s connectome to support functional transitions, independent of transient baseline functional fluctuations (Cui et al., 2020). The target state likewise does not reflect absolute neural activation but instead represents a canonical pattern in which nodes belonging to a given intrinsic connectivity network are set to be uniformly more active relative to all other regions. This simplification permits the estimation of the relative energetic cost required to engage each network from a common baseline under the same model constraints (Cui et al., 2020; He et al., 2022).

#### 2.3.3 Network controllability

To quantify the control-theoretic properties of each brain region, we computed average controllability and modal controllability from the normalized structural connectivity matrix A (Gu et al., 2015). Average controllability reflects a node’s ability to steer the system into nearby, easily reachable states with minimal control energy. It is computed analytically using the real Schur decomposition of A. Let $A=UTU^{T}$, where U is an orthonormal matrix and T is upper triangular with eigenvalues of A on the diagonal. The average controllability of node i is then defined as the sum

$$AC_{i}=\;\sum_{j=1}^{N}\frac{u_{ij}^{2}}{1-\;\lambda_{j}^{2}},\;$$

where $u_{ij}$ is the element in row i, column j of U, and $\lambda_{j}$ is the j-th eigenvalue of A.

Modal controllability, in contrast, captures a node’s ability to steer the system into difficult-to-reach modes—that is, those associated with slow, persistent dynamics. It is defined as

$$\emptyset_{i}=\;\sum_{j=1}^{N}\left(1-\;\lambda_{j}^{2}\right)u_{ij}^{2},\;$$

where $\lambda_{j}$ denotes the eigenvalues of the system matrix (via Schur decomposition) and $u_{ij}$ are elements of the corresponding orthonormal eigenvector matrix *U*. Nodes with high modal controllability tend to be influential in accessing dynamic configurations that require substantial reconfiguration of brain activity (Wu et al., 2024).

### 2.4 Normative modeling of control energy across canonical brain networks: Estimating brain age gap (BAG)

We implemented a normative modeling framework (Rutherford et al., 2022) to measure brain network control metric deviations across participants after adjusting for age-related demographic and lifestyle variables. First, we adjusted the control energy values for age-independent demographic and lifestyle covariates using ordinary least squares regression analysis. The covariates included sex (binary), smoking status, imaging center, household income, alcohol consumption frequency, educational attainment, ethnicity (White vs. non-White), Townsend Deprivation Index quintile, and “unknown/prefer-not-to-answer” categories. The adjusted control metric values obtained from these regressions served as inputs for subsequent normative modeling.

In the second step, we applied Bayesian linear regression (BLR) to characterize normative age-related patterns in the adjusted control energy metrics. This approach allows the identification of the expected age-based evolution of each metric in populations and detects individuals whose results deviate substantially from these expected values. The analysis used cubic B-splines with five degrees of freedom to model nonlinear age relationships. We trained distinct BLR models that used residual control values as outcomes while utilizing spline-expanded age as the predictive variable (Rutherford et al., 2022). We used 10-fold cross-validation to evaluate model performance. We performed the modeling and scoring using the PCNtoolkit, an open-source Python library developed for neuroimaging-based normative modeling (Rutherford et al., 2022). The age-related trajectories of network control energy across eight large-scale brain networks are described in Supplementary Figure S1.

We also computed network-specific BAGs for eight canonical brain networks. BAG was defined as the predicted age using cubic B-splines with five degrees of freedom minus the observed chronological age, expressed in years. To reduce potential age-related bias and account for regression to the mean effects, we fit the model with BAG as the dependent variable and chronological age as the predictor; the residuals from this model were taken as age-corrected BAG values, which were used for all subsequent statistical analyses.

### 2.5 Meta-analytic mapping: Cognitive states associated with MetS

We analyzed brain network control metrics and their cognitive functions relationships using functional MRI (fMRI) activation maps from Neurosynth (Yarkoni et al., 2011), which aggregates data from thousands of published neuroimaging studies. Although UKB also provides cognitive measures, they are limited to a few general domains. Furthermore, while estimates of control energy across canonical networks can capture large-scale energy costs, they lack the specificity to explain how individual cognitive functions are affected across overlapping or distributed networks. In contrast, our approach leverages meta-analytic activation maps to define a broad spectrum of cognitive states, allowing us to explore how MetS affects transition energy in a more comprehensive and functionally meaningful cognitive space. Our analysis included 123 cognitive activation maps that corresponded to experimentally validated cognitive domains such as attention, memory, language, and reasoning. These terms were selected from the Cognitive Atlas (Poldrack et al., 2011), an open ontology in the field of cognitive science containing a comprehensive list of neurocognitive terms; they were previously used by Luppi et al. (2024) and were adopted in this study as well. The 123 terms are listed in Supplementary Table S3.

We constructed spatial representations of each cognitive state as 216-dimensional brain vectors, consistent with our hybrid parcellation scheme combining the Schaefer 200-region cortical atlas and the Melbourne Subcortex Atlas (Schaefer et al., 2018). We used the Neuroimaging Meta-Analysis Research Environment and Neurosynth database to conduct a broad-scale meta-analysis to derive cognitive state spatial representations (Salo et al., 2023). The multilevel kernel density chi-square contrast of studies related to a term versus unrelated studies was conducted with a minimum of five studies per contrast (Taebi et al., 2022). We quantified the meta-analytic likelihood of activation given the cognitive term using z-maps saved as Neuroimaging Informatics Technology Initiative (NIfTI) volumes in Montreal Neurological Institute (MNI) space (1 mm resolution). The spatial representations used were volumetric association test maps, which represent the statistical likelihood that a specific term is mentioned in a study given the presence of activation at a particular voxel. These reverse-inference z-score maps provide a probabilistic measure of the relationship between regional brain activity and psychological processes. To account for multiple comparisons, the voxel-wise z-score maps were corrected using the false discovery rate (FDR). Each parcel contained the z-score average of activation that indicated the probability of regional activity linking to its corresponding cognitive process. The z-score values from each map were averaged to produce 216-dimensional brain space vectors. The cognitive term vectors contained spatial activation patterns that corresponded to each cognitive process.

To quantify how MetS-related alterations in network control energy impact distinct cognitive domains, we derived a cognitive vulnerability score for 123 cognitive states. This score was calculated by weighting the spatial activation patterns of each meta-analytic map by the absolute effect sizes (Cohen’s *d*) obtained from the MetS versus non-MetS comparisons across the networks showing significant energetic deviations. This binary contrast was chosen to align with clinical diagnostic standards and provide a robust effect-size weight for the meta-analytic map. This standardized metric allows for a ranking of functional domains based on their theoretical susceptibility to the reshaped energetic landscape observed in participants with metabolic syndrome.

These meta-analytic maps represent the theoretical functional topographies of specific cognitive states. We independently validated the behavioral relevance of these energetic findings by comparing them with the actual cognitive performance scores recorded from the UKB cohort participants. To this end, we utilized linear regression models to examine the relationships between MetS diagnosis and seven discrete UKB cognitive tasks, ensuring that all models were adjusted for the same demographic and lifestyle covariates applied in our primary analyses.

### 2.6 Spatial colocalization of network controllability deviations with PET and cell-type gene expression

Group differences were evaluated with parcel-wise two-sample t-tests, yielding *t*-maps for average and modal controllability deviations. To examine the biological underpinnings of these deviations, we employed the NiSpace toolbox (Lotter & Dukart, 2024; Lotter et al., 2023, 2024) to assess spatial colocalization with (i) PET receptor distribution maps, largely accessed via neuromaps (Markello et al., 2022) and (ii) cell type-specific gene expression maps derived from the Allen Human Brain Atlas (Hawrylycz et al., 2012; Markello et al., 2021). This spatial enrichment approach was motivated by the need to anchor NCT-derived energetic metrics within an established neurobiological context. Specifically, PET receptor distribution maps allow identification of neurotransmitter systems that may mediate altered network controllability in the context of metabolic dysfunction, while cell type-specific gene expression maps provide insight into whether the spatial heterogeneity of network-level energetic costs is associated with specific cellular populations—such as neurons or inflammation-linked glial cells—that are plausibly affected by MetS-related white matter compromise and its propagating effects on connected brain regions.

We utilized the neuromaps-curated repository (Markello et al., 2022) to obtain distribution maps for diverse neurotransmitter receptors and transporters. To ensure cross-modal comparability, all PET maps were transformed into the MNI152 coordinate system using standardized neuromaps workflows, ensuring that functional signals were aligned with anatomical templates. Also, spatial gene expression maps were derived from the Allen Human Brain Atlas, which provides high-resolution postmortem transcriptome data from six donors (Markello et al., 2021). We focused on regional signatures for non-neuronal cells (astrocytes, endothelial cells, microglia, oligodendrocytes) and 16 neuronal clusters (Ex1–8 and In1–8). We selected genes with a mean inter-donor Spearman correlation exceeding 0.1 to ensure spatial consistency. These data were preprocessed via spatial transformation to the MNI152NLin2009cAsym template (2 mm isotropic), brain extraction, and intensity scaling within a range of 10^–6^ to 1. Prior to statistical analysis, all continuous maps were parcellated using a combined Schaefer 200-region cortical atlas with the Melbourne Subcortex Atlas. This reduced the dimensionality to a common set of nodes and shared by our *t*-maps for controllability deviations. The associations between controllability deviations and biological maps were quantified using Spearman’s rank correlation coefficients. To ensure statistical rigor, we implemented two complementary null models: (i) spatial permutation of input maps using spin tests and Moran spectral randomization (Vos de Wael et al., 2020) (*n* = 10,000) to account for spatial autocorrelation and (ii) random resampling of gene sets matched for size to control for gene set bias. Associations were retained only if they survived both null models.

### 2.7 Statistical analysis

We used NumPy (v. 1.26.4), Pandas (v. 2.3.0), SciPy (v. 1.10.1), Statsmodels (v. 0.14.4), and Scikit-learn (v. 1.2.2) libraries in Python to perform the analyses. Continuous demographic and clinical variables are presented as means ± standard deviation. The overall trajectory of network alterations across the MetS spectrum was tested using linear mixed-effect models. To capture potentially nonlinear progression, we further modeled these trajectories using cubic splines. We compared network activation energy, computed from NCT, among MetS scores using one-way analysis of variance (ANOVA) with Tukey’s honestly significant difference (HSD) post hoc tests, and between MetS diagnosis groups (present vs. absent) using independent-sample t-tests and Cohen’s *d* effect sizes. We examined the relationships between activation energy and MetS score, as well as six individual metabolic indicators, using linear regression models controlling for age, sex, education, ethnicity, smoking, alcohol use, and socioeconomic status (Townsend Deprivation Index). The metabolic indicators and brain network activation energies were further explored using partial least squares correlation (PLSC) analysis, with bootstrap resampling (5,000 iterations) for reliability and permutation testing (5,000 iterations) for statistical significance (Krishnan et al., 2011). Statistical significance was defined as two-sided α = 0.05, with Benjamini–Hochberg FDR correction, where appropriate.

## 3 Results

### 3.1 Progressive metabolic, demographic, and cognitive changes across MetS severity levels

Greater MetS severity was associated with worsening metabolic health and cognitive performance (Supplementary Table S4). From scores 0 to 5, we observed increased mean age (51.78 ± 7.18 vs. 57.12 ± 6.62 years) and waist circumference (78.35 ± 8.71 to 104.68 ± 11.05 cm). Moreover, HbA1c (33.29 ± 2.94 to 46.02 ± 8.52 mmol/mol), SBP (118.20 ± 8.23 to 149.65 ± 14.91 mmHg), and triglyceride (1.02 ± 0.31 to 2.99 ± 1.17 mmol/L) level rose steadily, while HDL decreased (1.68 ± 0.35 to 1.03 ± 0.17 mmol/L).

Cognitive performance scores from the UKB cohort exhibited concordant downward trends across MetS severity levels (Supplementary Fig. S2). We evaluated the cross-sectional relationship between MetS score and performance across seven cognitive tasks. To facilitate direct comparison between cognitive tasks, all scores were standardized to z-scores. For measures where higher raw values indicate slower performance (TMT-A and TMT-B), scores were inverted such that lower z-scores consistently represented poorer cognitive function. These results revealed a consistent and statistically significant negative association between MetS score and all cognitive performance (all *P* < 0.001). The magnitude of the association was most pronounced for visual memory and working memory. Specifically, each 1-unit increase in MetS score was associated with a standardized decline of the PAL (β = -0.06, 95% CI [-0.07, -0.05], *P* < 0.001) and BDS test (β = -0.05, 95% CI [-0.06, -0.04], *P* < 0.001). The participants’ demographic and lifestyle characteristics are detailed in Supplementary Table S4 and were included as covariates.

### 3.2 Relationships between activation energy and BAG of eight canonical brain networks and MetS score

To ensure the reliability of the network-specific BAG estimates, we evaluated the performance of the normative models using 10-fold cross-validation (Supplementary Table S5). The models demonstrated statistically significant predictive accuracy across all eight canonical networks (all *P* < 0.001). The models for CONT (Spearman’s correlation ρ = 0.38) and DMN (ρ = 0.37) showed the highest alignment with chronological age. The mean absolute calibration error remained low across all networks (range: 0.013–0.022), indicating that the energetic cost deviations were precisely estimated relative to the normative aging trajectory.

MetS diagnosis was associated with significant activation energy differences in six of the eight networks (Table 1). Comparisons between the MetS and non-MetS groups showed the largest elevation for SUB (Cohen’s *d* = 0.22, 95% confidence interval (CI) [0.19, 0.25]; *P* < 0.001), followed by DAN (*d* = 0.11, 95% CI [0.18, 0.14]; *P* < 0.001) and SMN (*d* = 0.05, 95% CI [0.02, 0.08]; *P* = 0.002), while VIS showed reduction in the MetS group (*d* = -0.15, 95% CI [-0.18, -0.12]; *P* < 0.001).

**Table 1.** Group differences in activation energy by MetS diagnosis.

Network	Cohen’s d (95% CI)	FDR-adjusted *P* value
CONT	0.04 (0.01 – 0.07)	0.007*
DMN	0.01 (-0.02 – 0.04)	0.47
DAN	0.11 (0.08 – 0.14)	< 0.001*
LIM	-0.03 (-0.06 – -0.001)	0.04*
VAN	0.03 (-0.01 – 0.06)	0.11
SMN	0.05 (0.02 – 0.08)	0.002*
SUB	0.22 (0.19 – 0.25)	< 0.001*
VIS	-0.15 (-0.18 – -0.12)	< 0.001*

* FDR-adjusted *P* < 0.05.

Note: FDR, false discovery rate; CONT, control network; DMN, default mode network; DAN, dorsal attention network; LIM, limbic network; VAN, ventral attention network; SMN, somatomotor network; SUB, subcortical network; VIS, visual network.

**Fig. 3.**
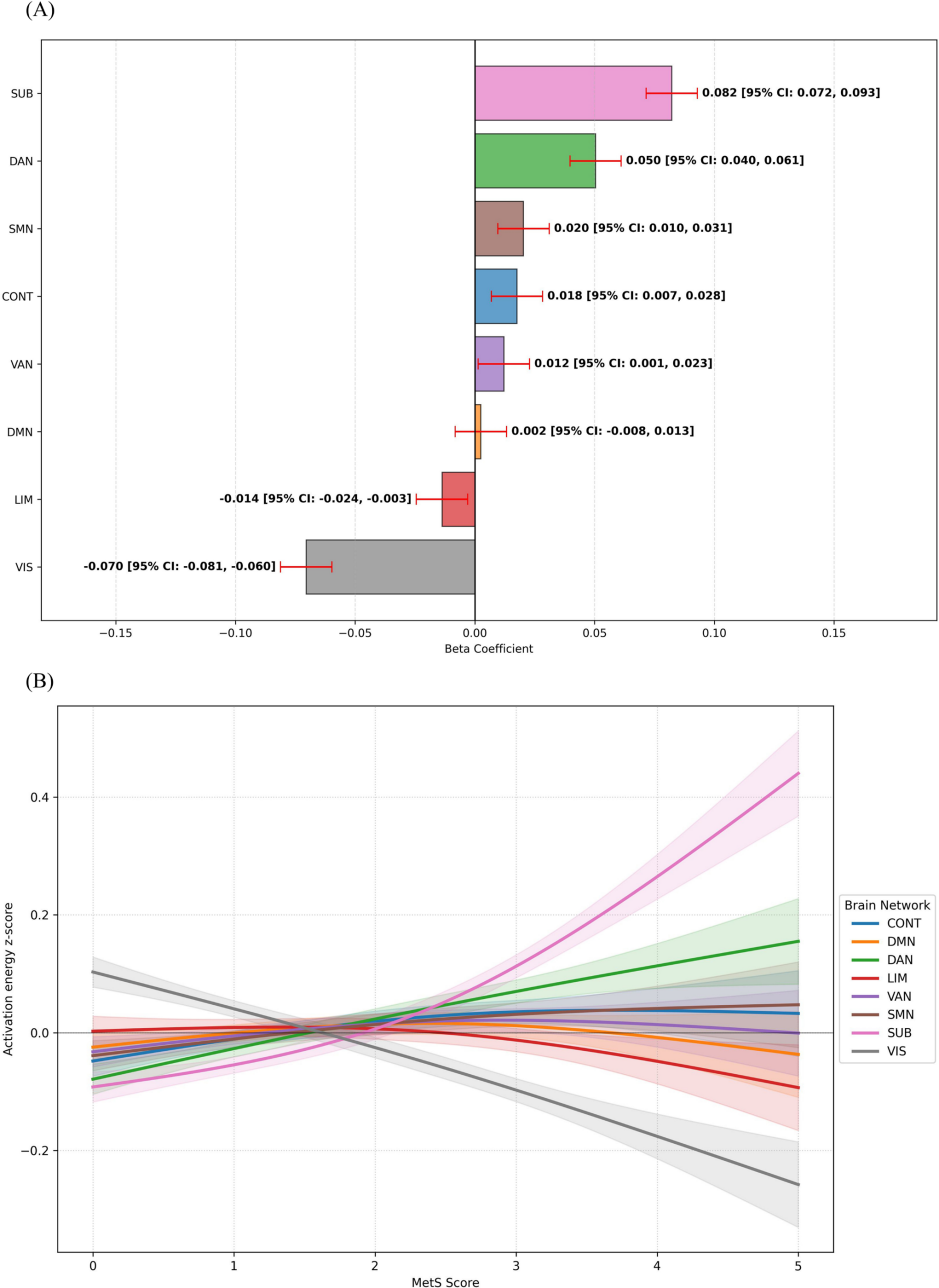
Relationships between MetS score and activation energy across brain networks. (A) Regression coefficients from linear mixed-effect model showing the standardized effect of MetS score on activation energy for each network. (B) Trajectories of network activation energy across MetS score range modeled using cubic splines. Shaded bands indicate 95% confidence intervals. CONT, control network; DMN, default mode network; DAN, dorsal attention network; LIM, limbic network; VAN, ventral attention network; SMN, somatomotor network; SUB, subcortical network; VIS, visual network; MetS, metabolic syndrome.

To test the relationship between continuous MetS scores and network energetics, we employed linear mixed-effect models (Fig. 3A). These analyses confirmed that higher MetS severity was positively associated with the activation energy required for SUB (β = 0.08, 95% CI [0.07, 0.09]; *P* < 0.001), DAN (β = 0.05, 95% CI [0.04, 0.06], *P* < 0.001), SMN (β = 0.02, 95% CI [0.01, 0.03]; *P* < 0.001), and CONT (β = 0.02, 95% CI [0.01, 0.03]; *P* < 0.001). Conversely, a negative association was found for VIS (β = –0.07, 95% CI [-0.08, -0.06]; *P* < 0.001). To further characterize these trends across the discrete metabolic symptom counts, we modeled the trajectories using cubic splines (Fig. 3B). This approach revealed a steady, graded increase in SUB energy and a consistent decrease in VIS energy as the MetS score progressed from 0 to 5.

To address the potential dependence of our findings on spatial resolution, we performed sensitivity analyses using the Schaefer 200-, 500-, and 1,000-parcel parcellations (Supplementary Fig. S3). These results showed no significant differences in regression coefficient between MetS score and activation energy z-score were observed between the 200 and 500 resolutions (All *P* > 0.05). High Pearson correlations (mean r = 0.84) further confirmed the spatial stability of these metabolic effect sizes. While the 1,000-parcel resolution showed attenuated coefficients in certain networks, likely due to increased signal sparsity and reduced signal-to-noise ratios in finer parcels, the overall patterns remained consistent.

**Fig. 4.**
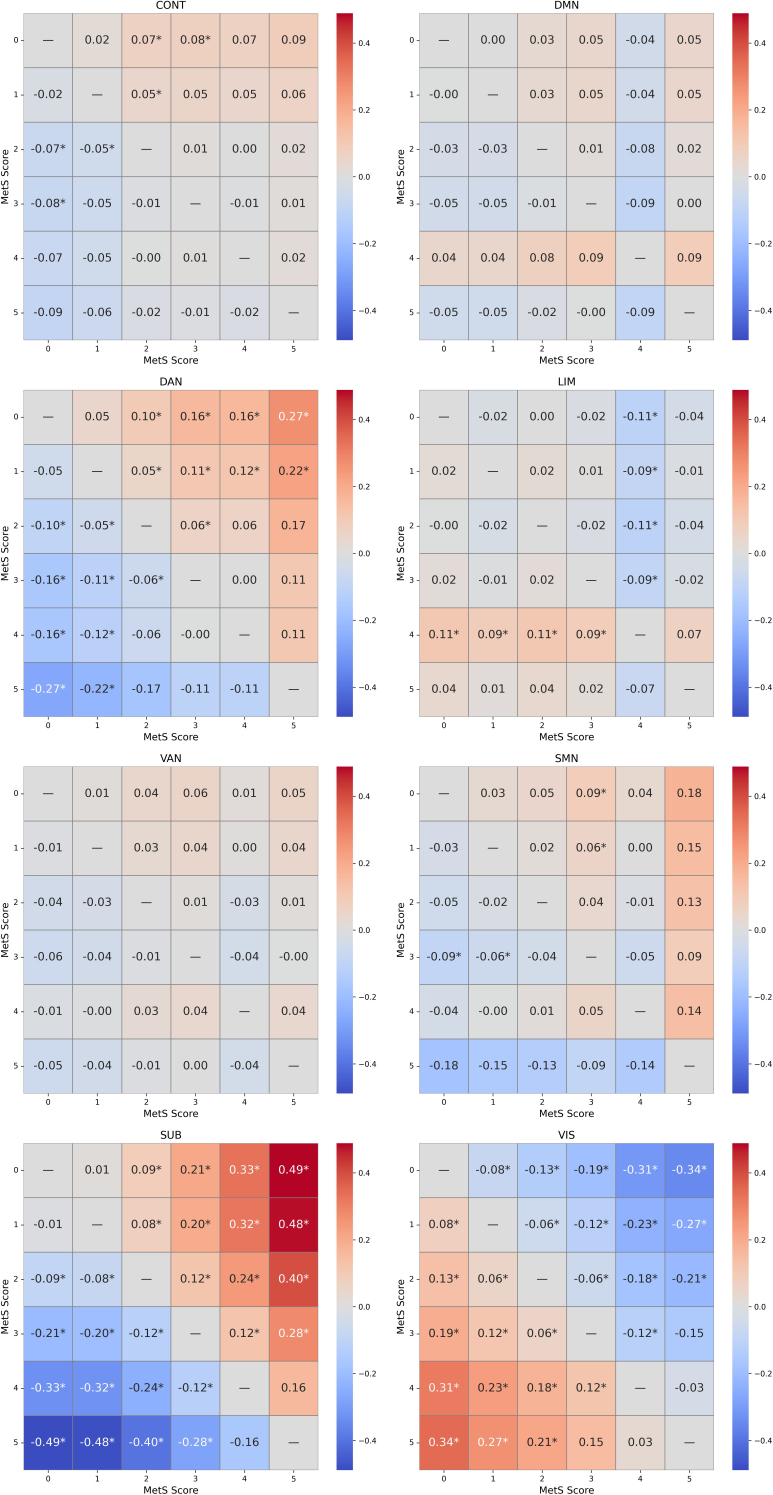
Pairwise differences in activation energy across MetS score groups for each brain network. Each panel represents a difference matrix of activation energy (z-score) between MetS score groups for a specific brain network, derived from post hoc analysis following the omnibus ANOVA. Post hoc comparisons were performed using Tukey’s HSD test to account for multiple comparisons. The rows and columns represent the number of metabolic syndrome criteria met by participants (MetS score 0 to 5). Each cell displays the estimated mean difference between the row group and the column group, and asterisks (*) indicate statistically significant differences surviving Tukey’s HSD correction (*P* < 0.05). Color intensity indicates both the direction and magnitude of the difference: red represents higher activation in the row group than in the column group, while blue represents lower activation energy. CONT, control network; DMN, default mode network; DAN, dorsal attention network; LIM, limbic network; VAN, ventral attention network; SMN, somatomotor network; SUB, subcortical network; VIS, visual network; MetS, metabolic syndrome.

ANOVA showed significant score-related differences in all networks except for VAN (*P* = 0.06), with the largest F-statistics for SUB, VIS, and DAN (all *P* < 0.001) (Supplementary Table S6). Post hoc analyses using Tukey’s HSD test quantified the magnitude of these shifts, with the estimated mean differences of z-score visualized in Figure 4. Specifically, SUB exhibited a progressive increase in energy cost at nearly every score step, with a substantial cumulative difference of approximately 0.49 between scores 0 and 5. In contrast, VIS showed consistent reductions, reaching a maximum difference of -0.34 between score groups 0 and 5. DAN differences emerging between score 0–1 and ≥ 2, with the largest group difference reaching 0.27. CONT and SMN differences appeared between lower (0–1) and mid-range (2–3) scores. We observed LIM reductions at score 4, and DMN differences were significant only in omnibus testing.

**Fig. 5.**
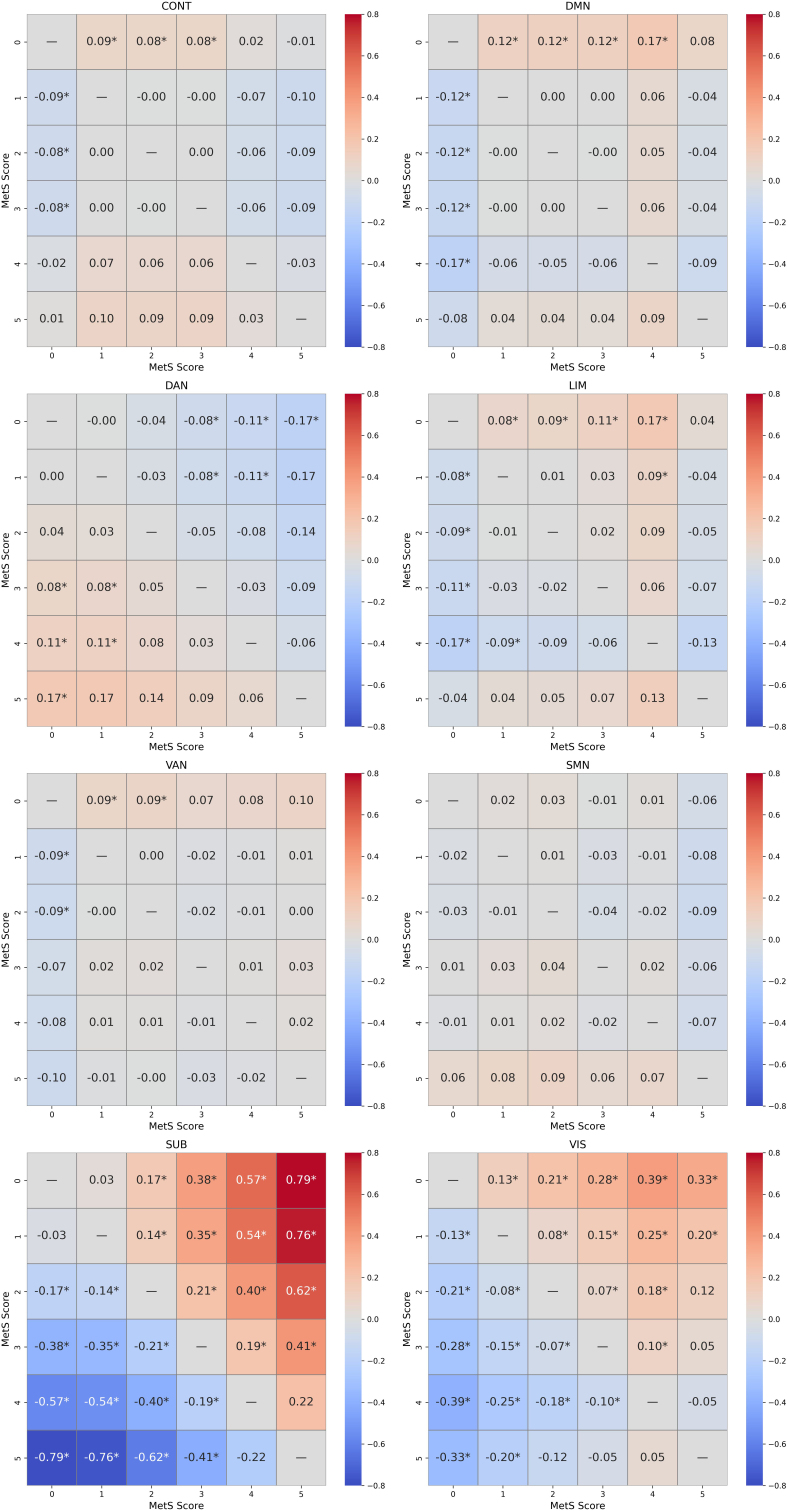
Pairwise differences in network-specific BAG across MetS score groups. Each panel shows the mean BAG difference (in years) between pairs of MetS score groups for a specific brain network. Post hoc comparisons were performed using Tukey’s HSD test to account for multiple comparisons. Cells show the estimated mean difference between groups, and asterisks (*) denote statistically significant differences (*P* < 0.05). Positive values (red) indicate that the row group exhibited an older-appearing brain relative to the column group, whereas negative values (blue) indicate a younger-appearing brain. CONT, control network; DMN, default mode network; DAN, dorsal attention network; LIM, limbic network; VAN, ventral attention network; SMN, somatomotor network; SUB, subcortical network; VIS, visual network; MetS, metabolic syndrome.

The BAG trajectories mirrored the activation energy patterns (Fig. 5). SUB showed the steepest increase, with differences emerging at score 2 (+0.17 years by score 0, 95% CI [0.08, 0.26]; *P* < 0.001) and reaching +0.79 years at score 5 (95% CI [0.48, 1.10]; *P* < 0.001). VIS BAG rose from +0.13 years at score 1 (95% CI [0.08, 0.18]; *P* < 0.001) to +0.39 years at score 4 (95% CI [0.30, 0.47]; *P* < 0.001). DMN (+0.17 years by score 4, 95% CI [0.06, 0.29]; *P* < 0.001) and LIM (+0.17 years by score 4, 95% CI [0.08, 0.27]; *P* < 0.001) increases were smaller. DAN changes were modest and SMN differences were non-significant.

We further conducted a combination-specific analysis to determine whether the specific composition of MetS, rather than just MetS score, influenced brain dynamics. For individuals with identical MetS scores ranging from 1 to 3, the presence or absence of specific components led to significant variations in activation energy, particularly within SUB and VIS. Elevated waist circumference was identified as the most critical driver of these differences. This compositional influence was not observed in participants with a MetS score of 4, where individual variation in the combination of metabolic criteria did not yield statistically significant differences in network energetics (Supplementary Tables S7 and S8).

### 3.3 Relationship between the activation energies of eight canonical brain networks and metabolic indicators

Standardized regressions (Supplementary Fig. S4) identified waist circumference as the most consistent correlate, which was positively associated with SUB (β = 0.13, 95% CI [0.12, 0.15]; *P* < 0.001) and negatively with VIS (β = –0.10, 95% CI [-0.11, -0.09]; *P* < 0.001). HDL was positively associated with VIS (β = 0.04, 95% CI [0.02, 0.05]; *P* < 0.001) and negatively with other networks. HbA1c was positively associated with SUB (β = 0.06, 95% CI [0.05, 0.07]; *P* < 0.001) and negatively associated with VIS (β = -0.02, 95% CI [-0.03, -0.01]; *P* < 0.001) and LIM (β = -0.02, 95% CI [-0.03, -0.01]; *P* = 0.003). In multivariable models (Supplementary Fig. S5), waist circumference remained the strongest predictor for SUB (β = 0.13, 95% CI [0.12, 0.14]; *P* < 0.001) and VIS (β = –0.10, 95% CI [-0.11, -0.08]; *P* < 0.001).

**Fig. 6.**
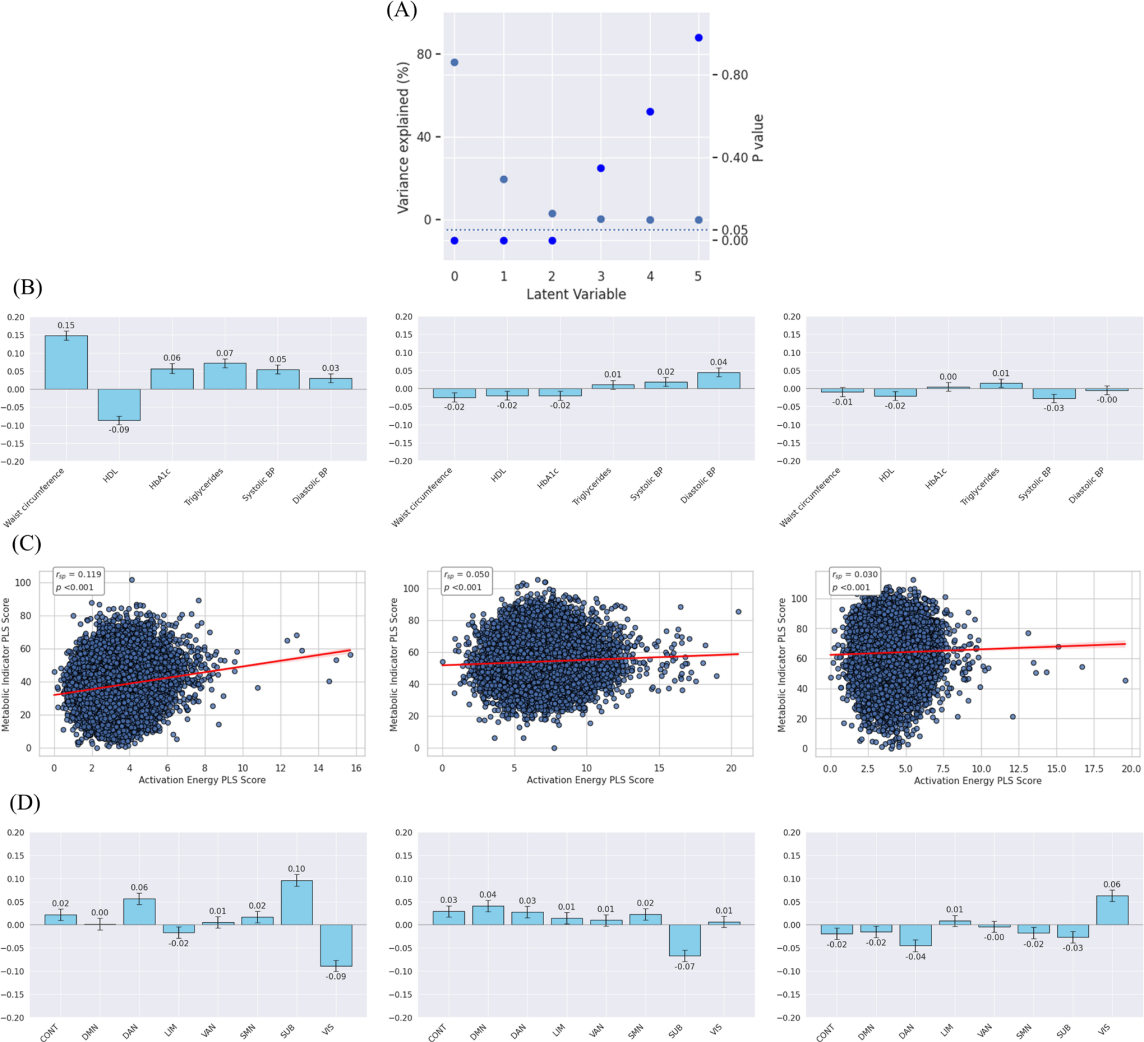
Multivariate associations between metabolic indicators and brain network activation energy via PLSC. Results from PLSC analysis linking metabolic indicators to brain network activation energy (z-scores) estimated using network control theory. (A) Variance explained by each latent variable, with associated permutation-based *P* values. The first three latent variables are statistically significant. (B) Bootstrap-estimated covariance profiles of metabolic indicators for significant latent variables, reflecting their relative contributions to the multivariate brain–metabolic association pattern. (C) Scatterplots showing the correlation between subject-level PLSC-derived activation energy scores and metabolic indicator scores for each significant latent variable. (D) Corresponding covariance profiles of network activation energy features, indicating which brain systems contribute most to the multivariate association. CONT, control network; DMN, default mode network; DAN, dorsal attention network; LIM, limbic network; VAN, ventral attention network; SMN, somatomotor network; SUB, subcortical network; VIS, visual network; HDL, high-density lipoprotein cholesterol; BP, blood pressure; PLS, partial least squares.

PLSC analysis identified three significant latent variables (LVs) linking metabolic indicators with network activation energy (Fig. 6). The first LV (77.8% of the shared variance; *P* < 0.001) reflected an adverse metabolic profile: positive loadings for waist circumference (0.15, 95% CI [0.14, 0.16]), triglycerides (0.07, 95% CI [0.06, 0.08]), and HbA1c (0.06, 95% CI [0.04, 0.07]), and a negative loading for HDL (–0.09, 95% CI [–0.10, –0.07]). This profile was associated with higher activation energies in SUB (0.10, 95% CI [0.08, 0.11]), DAN (0.06, 95% CI [0.04, 0.07]), and CONT (0.02, 95% CI [0.01, 0.03]), and lower energy in VIS (–0.09, 95% CI [–0.10, –0.08]). The second and third LVs explained less variance and coherence.

To examine potential age-related variation in these metabolic–network associations, we repeated the PLSC analysis separately for participants in their 40s, 50s, and 60s. The results of these age-stratified PLSC analyses showed weaker metabolic network coupling in the 40s, which increased in the 50s and 60s (Supplementary Figs. S6–S8). Waist circumference and HDL contributions strengthened with age, along with rising SUB and DAN loadings, suggesting more pronounced effects of metabolic profiles on subcortical and attentional networks in later decades.

### 3.4 MetS-sensitive cognitive states based on network control alterations

Vulnerability scores were calculated as a weighted sum of reverse-inference activation across six brain networks (SUB, VIS. DAN, SMN, CONT, and VAN; Table 1) that showed significant MetS-related differences in activation energy. The results of the meta-analytic mapping revealed the highest vulnerability scores for memory retrieval processes (retrieval, encoding, memory), spatial cognition (navigation), and mental imagery tasks (Table 2). Behaviorally, MetS diagnosis was associated with reduced verbal memory (PAL: β = –0.09, 95% CI [-0.11, -0.06]; *P* < 0.001), working memory (BDS: β = –0.07, 95% CI [-0.10, -0.04]; *P* < 0.001), and processing speed (SDS: β = –0.06, 95% CI [-0.09, -0.03]; *P* < 0.001) (Supplementary Table S9). The effects of simple processing speed (TMT-A), executive function (TMT-B), and nonverbal reasoning (MPC) were small and not statistically significant. In addition, changes in cognitive performance based on the MetS score showed a similar trend (Supplementary Fig. S2).

**Table 2.** Top 10 cognitive states most vulnerable to MetS-related alterations in brain network control.

Cognitive state	Vulnerability score
Retrieval	0.90
Encoding	0.89
Memory	0.79
Navigation	0.75
Imagery	0.74
Anticipation	0.69
Pain	0.69
Learning	0.58
Action	0.56
Movement	0.56

These behavioral findings, particularly those in the memory and processing speed domains, partially overlapped with the network control-derived vulnerability profile. However, the cognitive map captured only a subset of the meta-analytic cognitive states, and network control estimates were model-based rather than direct neural measures. Thus, the behavioral results should be interpreted as complementary evidence rather than a complete validation of the brain-derived vulnerability estimates.

### 3.5 Colocalization of network controllability deviation with PET and cell-type gene expression

**Fig. 7.**
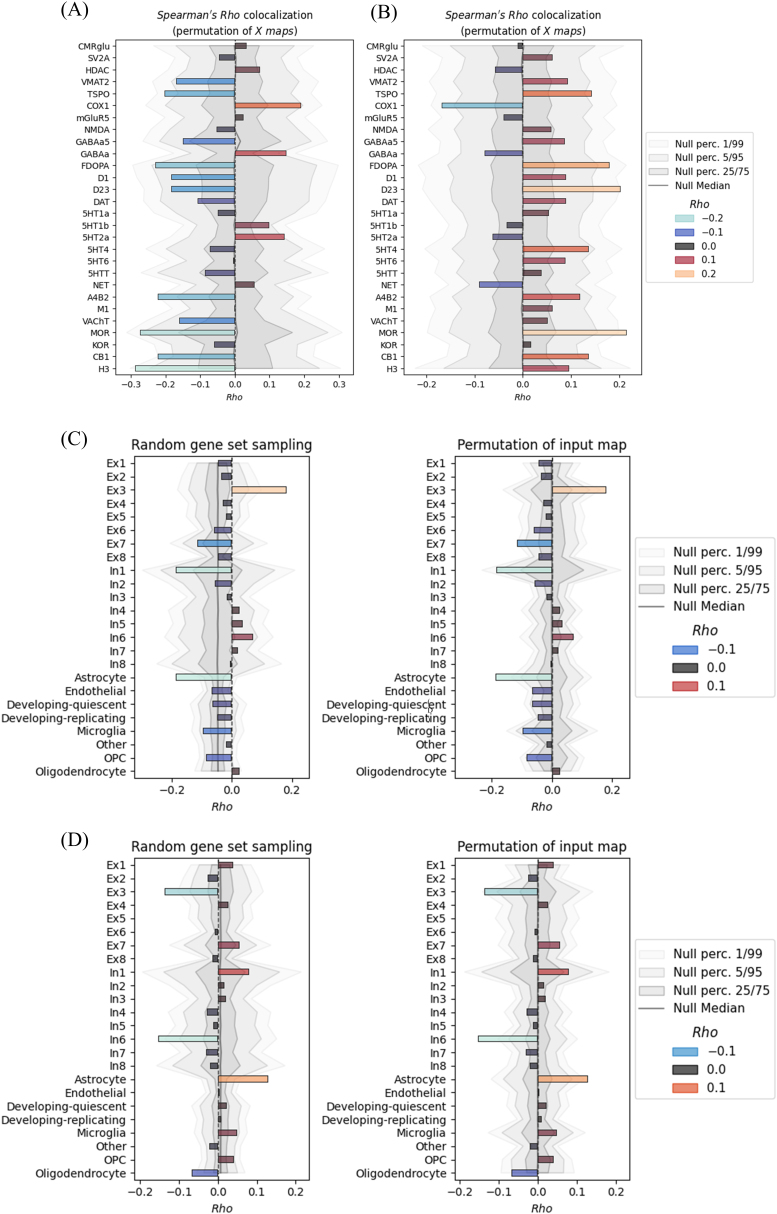
Spatial colocalization of controllability deviations with PET receptor maps and cell type-specific gene expression. To ensure statistical rigor, we implemented two null models: (i) spatial permutation via spin tests and Moran spectral randomization to account for spatial autocorrelation and (ii) size-matched gene set resampling to control for potential bias. Gray distributions indicate null models (1/99, 5/95, and 25/75 percentiles), with black lines denoting the null median. Bar colors indicate the direction and magnitude of Spearman’s *ρ*. (A) Spatial associations between average controllability deviations and in vivo PET receptor/transporter distribution maps. (B) Spatial associations between modal controllability deviations and PET receptor/transporter maps. (C) Spatial colocalization of average controllability deviations with cell type-specific gene expression maps from the Allen Human Brain Atlas. (D) Spatial colocalization of modal controllability deviations with cell type-specific gene expression.

Group difference statistical t-maps derived from normative modeling of average and modal controllability z-scores were tested for spatial colocalization. For PET receptor distribution map, average controllability deviations were significantly associated with γ-aminobutyric acid type A (GABA_A_) and serotonin 2A receptor (5-HT2A) distributions (Fig. 7A). Modal controllability deviations exhibited broader associations, colocalizing significantly with *μ*-opioid receptors (MOR), dopamine D2/3 receptors, cannabinoid CB1 receptors, presynaptic dopamine synthesis capacity (FDOPA), and translocator protein (TSPO; a marker of neuroinflammation) (Fig. 7B). For cell type-specific gene expression, average controllability deviations showed spatial correspondence with astrocytic and excitatory neurons type 3 (layer 4) gene expression patterns (Fig. 7C). Modal controllability deviations demonstrated a similar convergence on astrocytic and excitatory neuron type 3 distributions, while additionally exhibiting significant colocalization with inhibitory neuron type 6 (layers 4/5) (Fig. 7D).

## 4 Discussion

The results of this large, population-based neuroimaging study demonstrated that the metabolic cost of MetS is systematically embedded in network-level control energy profiles, particularly within subcortical and visual network. Across the full spectrum of MetS severity, activation energy of subcortical network increased progressively with each incremental MetS score, whereas activation energy of visual network decreased in a similarly graded fashion from one MetS score to the next higher score. Other networks displayed threshold effects, with changes emerging only beyond specific MetS score levels. These patterns suggest that some brain systems exhibit a gradual, dose–response vulnerability to metabolic stress, whereas others remain relatively resilient until a tipping point is reached (Machado-Fragua et al., 2022). The result of the BAG analysis reinforced these findings: subcortical and visual network showed a stepwise BAG increase with higher MetS scores, consistent with accelerated aging phenotypes, while other networks exhibited changes only at discrete transitions. Also, our multi-resolution analysis confirms that the identified relationships between metabolic risk and brain network controllability are not artifacts of a specific parcellation scheme. The high correlation between Schaefer 200 and 500 results suggests that moderate spatial resolutions capture stable physiological signals.

These results extend prior structural imaging studies, showing that MetS-related brain effects are not uniform but follow distinct network-specific trajectories that may be more informative than global brain-aging measures (Rashid et al., 2021). The progressive BAG increase observed in the present study may reflect not only demographic differences but also accelerated biological aging driven by cumulative metabolic cost. Owing to its high metabolic demand and vascular sensitivity, the brain is particularly susceptible to chronic metabolic stress, which may accelerate structural and functional deterioration beyond that expected from chronological aging alone. Also, given the subcortical network’s role in memory consolidation, motor coordination, and affective regulation, its premature aging may contribute to deficits in memory retrieval, spatial navigation, and mental imagery.

Network responses clustered into three categories: (i) gradual-change (SUB, VIS, DAN), (ii) critical-point (CONT, SMN, LIM), and (iii) relatively resilient (DMN, VAN) patterns. While MetS induces metabolic, vascular, and inflammatory stress throughout the brain, its impact is regionally heterogeneous. Resting-state fMRI studies in MetS populations reported reduced modularity and hyper-connectivity between core networks (Rashid et al., 2021). Our findings demonstrate that such changes are often nonlinear. Continuous increases in activation energy of subcortical and dorsal attention network may reflect cumulative processes such as insulin resistance, microvascular injury, and dopamine metabolism abnormalities. Conversely, the abrupt changes in control, somatomotor, and limbic network support the concept of turning points described in nonlinear aging models (Antal et al., 2025; Jordi & Isacson, 2024).

Among metabolic indicators, waist circumference emerged as the primary driver of network-level energy alterations, exerting the largest effects on subcortical and visual network, followed by smaller effects from dyslipidemia (elevated triglyceride and reduced HDL levels). The influence of central adiposity on network energetics increased with chronological age, indicating that the cumulative impact of worsening metabolic symptoms, as captured by higher MetS scores, progressively amplifies network vulnerability. Here, vulnerability refers to the increased energetic cost required to engage specific brain networks, which scales with the severity of the metabolic profile. From a preventive medicine standpoint, these findings support early, sustained management of visceral fat as a modifiable target to preserve brain efficiency and cognitive function. Mechanistically, excess central fat is linked to chronic inflammation, endothelial dysfunction, and insulin resistance, all of which impair cerebral perfusion and myelination, thereby increasing the input energy required to achieve a given neural state (Jais & Brüning, 2017; Trofimova et al., 2023). Our results support this hypothesis at the network level, showing that pathways integrating interoceptive, reward, and autonomic signals (SUB) become energetically costly, paralleling fluorodeoxyglucose PET (^18^F‑FDG PET)-based evidence of hyper‑metabolism in central‑obesity phenotypes (Pak et al., 2018). Conversely, the highly myelinated visual network becomes less excitable, consistent with synaptic downscaling (Cakir et al., 2024; Critchley & Harrison, 2013; Glasser & Van Essen, 2011). Limbic, ventral attention, and default mode network showed negligible or inconsistent shifts in the present study, aligning with the concept that these trans-modal systems benefit from redundant vascular supply and metabolic flexibility, rendering them resilient in mid-life but vulnerable in advanced pathology (Godbersen et al., 2023). Moreover, the observed network gradation mirrored the principal gradient of cortical microstructure and metabolism. Unimodal sensory networks (lower gradient) exhibited energy reductions, whereas trans-modal integrative networks (higher gradient) showed increases, consistent with large-scale models of hierarchical energy flow (Bernhardt et al., 2022; Huntenburg et al., 2018). The elevated activation energy in subcortical and dorsal attention network, alongside its reduction in visual network, likely reflects a reduced capacity to engage metabolically demanding neural states rather than alterations in routine, low-cost transitions.

By mapping effect sizes from MetS diagnosis contrasts onto 123 meta-analytically defined cognitive states, we identified memory retrieval, encoding, and other episodic memory processes as the most vulnerable functional domains. These domains rely on hippocampal–subcortical loops and frontoparietal attentional hubs, consistent with the circuits showing the strongest energy changes in our data (Spagna et al., 2021; Torres-Morales & Cansino, 2024). Cognitive testing provided indirect behavioral validation: we observed that individuals with higher metabolic cost performed worse on episodic memory (PAL), working memory (BDS), and processing speed (SDS) tasks, all of which depend on integrity of subcortical, dorsal attention, and visual network. Tasks dependent on more resilient networks, such as set-shifting and reasoning, were relatively spared, mirroring the smaller energetic perturbations in control network. Although these tests did not capture the full range of vulnerable cognitive states, the concordance between model-derived vulnerability and observed behavioral deficits provides evidence for the functional relevance of network control metrics.

This study demonstrates that z-scores in network controllability associated with MetS align spatially with distinct neurotransmitter systems and cell-type distributions. Deviations in average controllability showed significant colocalization with astrocytic and excitatory granule neuron gene expression, as well as with GABA_A_ and 5-HT2A receptor maps. These findings suggest that disruptions in baseline network control capacity are linked to astrocytic metabolic support and serotonergic modulation, consistent with prior evidence that astrocytes regulate neuronal excitability and energy supply (Naveed et al., 2025; Zhang et al., 2023). Furthermore, previous study has emphasized that excitatory pyramidal neurons, particularly in input layers, are strongly modulated by serotonergic and GABAergic systems (Ju et al., 2020), underscoring a plausible pathway through which astrocytic dysfunction and neurotransmitter imbalance may jointly constrain controllability. Modal controllability deviations exhibited broader associations, extending to inhibitory neurons subtype 6, as well as opioid, dopamine D2/3, CB1 receptor distributions, alongside FDOPA and TSPO maps. These findings converge with recent computational work by Luppi et al. (2024), who showed that the neuroanatomical distribution of MOR and dopaminergic receptors most effectively facilitates transitions between cognitive topographies. The overlap between our empirical results and these computational predictions suggests that the very receptor systems optimized to enable flexible cognitive transitions under normal conditions are also those in which deviations emerge in MetS. Overall, these results highlight a mechanism by which MetS perturbs controllability through astrocytic dysfunction, disrupted excitatory–inhibitory balance, and altered neuro-modulatory influences.

Clinically, these results advance the field in three ways. First, they establish NCT-derived activation energy as a sensitive intermediate phenotype for detecting subclinical metabolic effects before adverse impacts on neuronal integrity manifest as overt disruptions in the structural connectome. By focusing on network-level energetic costs, this approach provides an earlier window into the functional consequences of metabolic dysfunction than traditional structural connectivity measures alone. Second, they identify specific networks, particularly subcortical hubs, whose energetic cost scales with central adiposity, thus offering potential therapeutic targets. Finally, the findings support a mechanistic cascade in which visceral fat and dyslipidemia perturb subcortical energetics, propagate dysfunction to attentional and executive circuits, and manifest as domain-specific cognitive inefficiencies. Monitoring waist circumference and lipid profiles, even before full MetS criteria are met, could help identify and allow for timely intervention in individuals at risk for early network disruption.

This study has some limitations. First, the cross-sectional design prevented the determination of causality; therefore, longitudinal and interventional studies are needed to test reversibility. Also, the temporal gap between the baseline metabolic assessment and the follow-up neuroimaging scan. While we cannot fully account for fluctuations in MetS status during this period, metabolic markers have shown high intra-individual stability over time in the UKB cohort (Allen et al., 2020; Qureshi, Topiwala, et al., 2024). Furthermore, the use of baseline data may provide a more robust estimate of chronic exposure to metabolic dysfunction. Second, HbA1c was used as a proxy for glycemic control, limiting the resolution of glucose-related effects. Repeated fasting measurements or oral glucose-tolerance tests would sharpen the metabolic phenotype and allow mechanistic modeling of insulin resistance. Third, while this study controlled for major demographic and lifestyle factors, we did not explicitly account for metabolic-related medications or psychiatric comorbidities such as depression. Medications may mitigate some physiological effects of MetS, potentially leading to an underestimation of the structural–functional deficits. Future research utilizing longitudinal data should investigate how the initiation of treatment or the presence of affective disorders influences the energetic cost of brain state transitions over time. Fourth, the LTI model simplifies brain dynamics; incorporation of nonlinear control frameworks could refine energy estimates. Fifth, large sample sizes may yield statistically significant but small effect sizes. The majority of standardized β coefficients (< 0.10) are unlikely to translate directly into clinically meaningful deficits in isolation, which is expected given that cognitive performance and brain network integrity are influenced by a multitude of genetic, environmental, and lifestyle factors beyond metabolic health alone. Although we excluded participants with self-reported neurodegenerative disorders, the presence of subclinical neurological impairments in the remaining sample cannot be fully ruled out, and the majority of participants are assumed to have not yet reached clinical thresholds of neurological impairment. Nevertheless, these small subclinical deviations in network control energy may represent the earliest stages of energetic inefficiency—a physiological signature that precedes gross structural atrophy or overt clinical symptoms. Within a neural resilience framework, these subtle shifts may reflect a progressive loss of adaptive capacity that compounds over time under sustained metabolic cost. Future work should benchmark observed energetic deviations against longitudinal outcomes such as incident mild cognitive impairment, functional decline, or dementia conversion to confirm the clinical utility of these findings. Sixth, the cohort was restricted to midlife-early older adults. Network control energy responses to metabolic stress may differ in adolescents (where networks are still maturing) or in the oldest-old (where neurodegeneration predominates). While this age restriction limits the direct generalizability of the findings, the methodology has potential utility in other populations where metabolic and nutritional factors critically shape brain and cognitive development. For example, applying this framework to conditions including childhood and adolescent obesity, type 1 diabetes mellitus, or eating disorders (e.g., bulimia nervosa, binge eating disorder) could help elucidate how metabolic dysregulation interacts with neural maturation trajectories and cognitive function (Kansra et al., 2020). Seventh, while we utilized a large population-based sample to define our normative models, it is important to acknowledge that this normative group may not be entirely free of subclinical or clinical mental and physical conditions, including metabolic dysregulation. This represents an inherent challenge in population-scale normative modeling, where the healthy distribution may still encompass a degree of latent pathology. Finally, cognitive state mapping relied on meta-analytic, rather than task-evoked, neural patterns. To validate our inferences, future studies should acquire neuroimaging data as participants actively perform the corresponding cognitive tasks.

## 5 Conclusions

Metabolic dysfunction systematically reshapes the energetic demands of large-scale brain networks, accelerating brain aging and reducing cognitive ability. NCT-derived energy metrics offer modifiable, clinically relevant biomarkers linking metabolic health to brain network control energy and cognitive performance. Integrating these measures into precision prevention strategies, particularly those targeting central adiposity and dyslipidemia, may help preserve cognitive function across the lifespan.

## Supplementary Material

Supplementary Material

## Data Availability

Data originate from the UK Biobank and will be accessible contingent upon authorization from the UK Biobank (https://www.ukbiobank.ac.uk/). Code used for analysis is available on our GitHub repository (https://github.com/phs9416/UKB_MetS_NCT/).
