# Supplementary material for: Metabolic syndrome severity and the energetic cost of brain network transitions: A normative modeling study of accelerated brain aging
Source: Imaging Neurosci (Camb). 2026 Jun 22;4:IMAG.a.1282. doi: 10.1162/IMAG.a.1282 (PMC13288504; doi:10.1162/IMAG.a.1282)
Supplement: Supplementary Material [file IMAG.a.1282_supp.pdf]

Supplementary Figure S1 Age-related trajectories of network control energy across eight large-scale brain networks. (A) Each subplot shows the relationship between age and activation energy for one brain network, based on data from all participants. The scatter point represents individual participant. The solid black line denotes the overall population-level trend (median) estimated using cubic B-spline regression. The dashed black lines indicate the 5th, 25th, 75th, and 95th percentiles of the overall distribution, representing the normative range of variability. The colored lines depict median trajectories for subgroups stratified by MetS score. These plots enable the identification of deviations in network control energy, particularly in the context of brain aging. (B) Visualization of eight brain networks. CONT, control network; DMN, default mode network; DAN, dorsal attention network; LIM, limbic network; VAN, ventral attention network; SMN, somatomotor network; SUB, subcortical network; VIS, visual network; MetS, metabolic syndrome.

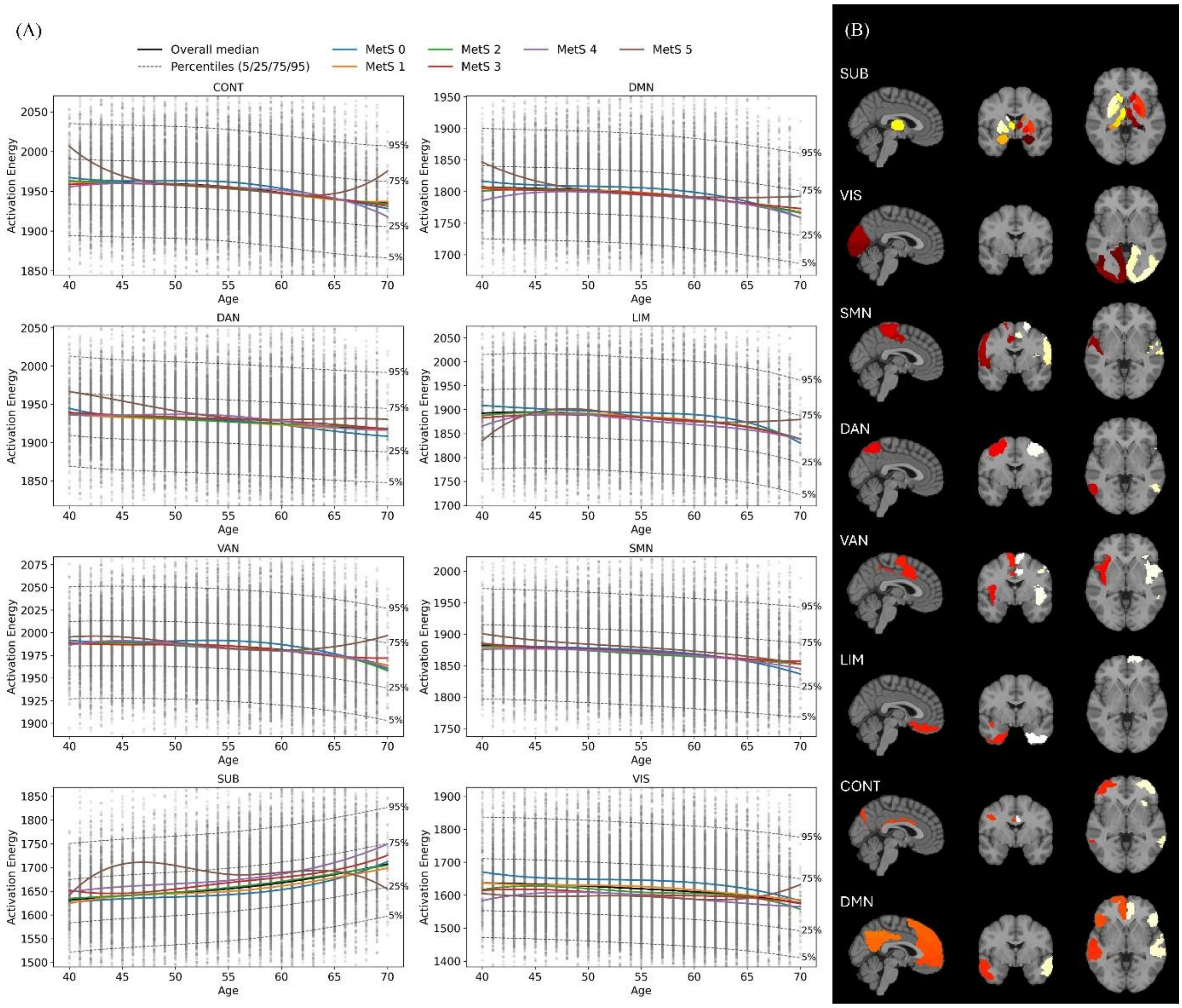

Supplementary Figure S2 Impact of MetS on cognitive function. (A) Cognitive performance trajectories across MetS score. Cognitive scores were converted to z-scores (mean = 0, standard deviation = 1) to enable direct comparison across metrics. For the trail making tests (TMT-A and TMT-B), scores were inverted such that a downward trend consistently represents worsening performance across all displayed variables. Shaded regions represent the 95% confidence intervals. (B) Forest plot showing the standardized coefficients from multivariable linear regression, adjusted for age, sex, and socioeconomic status. Error bars represent the 95% confidence intervals. CONT, control network; DMN, default mode network; DAN, dorsal attention network; LIM, limbic network; VAN, ventral attention network; SMN, somatomotor network; SUB, subcortical network; VIS, visual network; MetS, metabolic syndrome; TMT, trail making test; FI, fluid intelligence; BDS, backward digit span; SDS, symbol digit substitution; PAL, paired associate learning; MPT, matrix pattern completion.

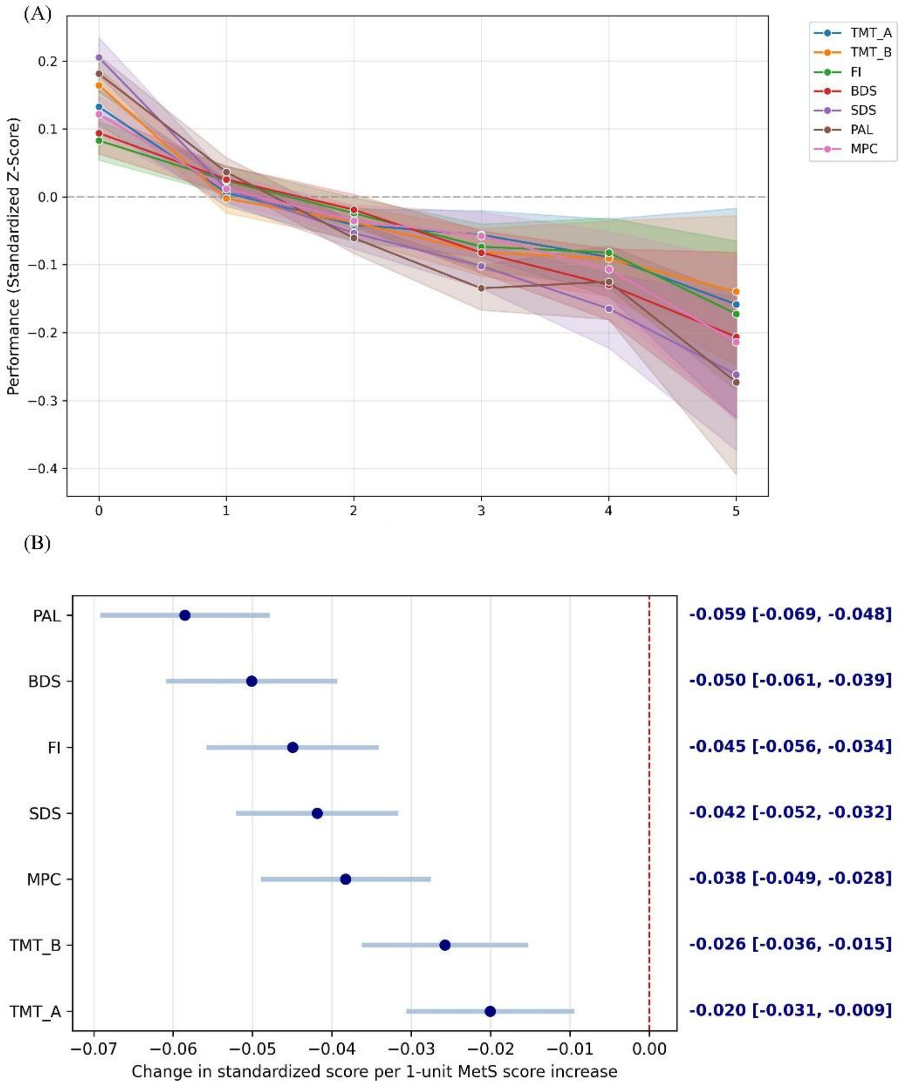

Supplementary Figure S3 Robustness analysis across spatial resolutions. (A) Pearson correlation matrix of network-level activation energy z-scores between different atlas pairs. (B) Comparison of regression coefficients for the relationship between MetS score and activation energy z-scores across Schaefer 200, 500, and 1,000 parcellations. Asterisks (*) indicate significant differences in effect sizes between resolutions (P < 0.05).

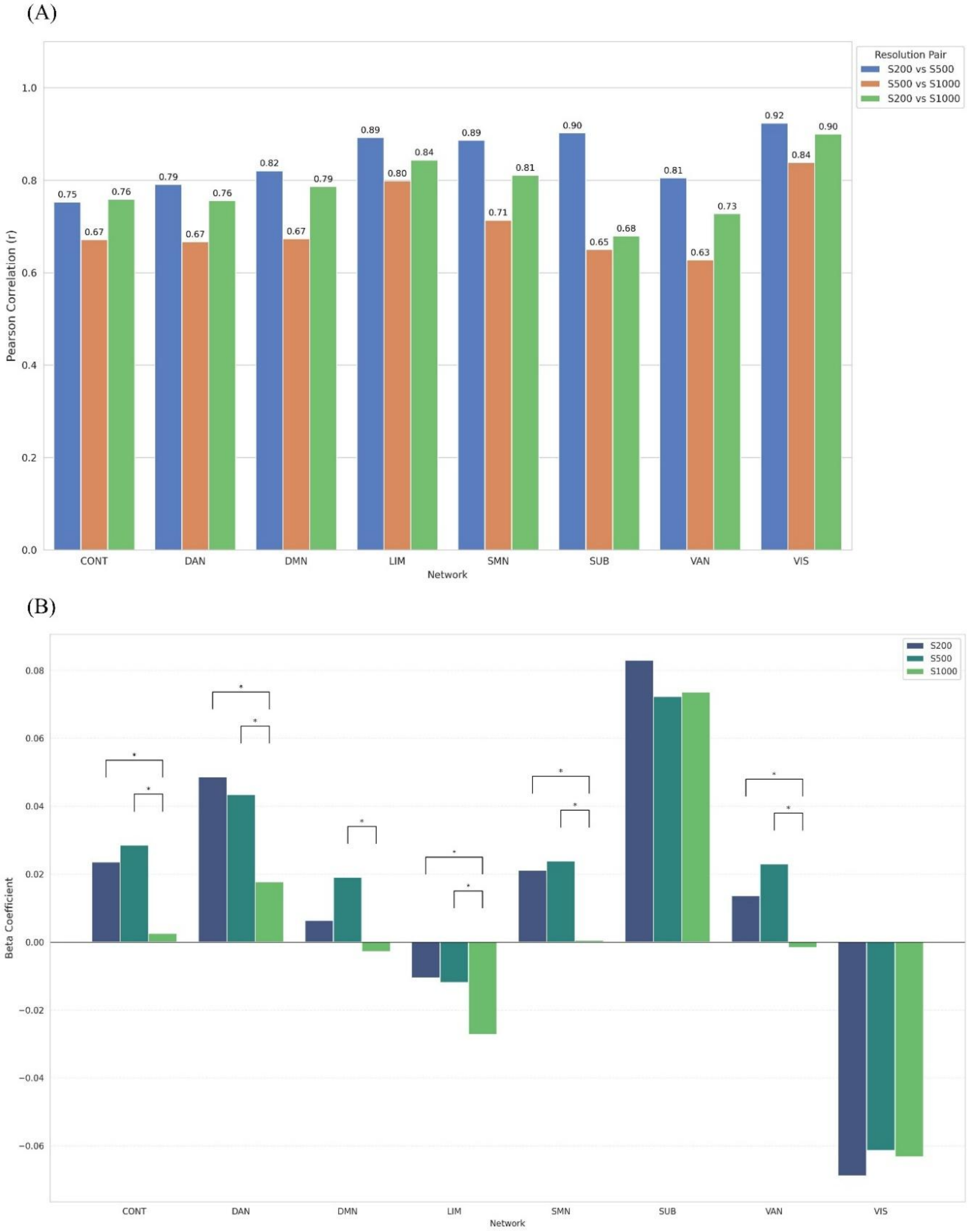

Supplementary Figure S4 Standardized regression coefficients between selected metabolic indicators and activation energy across major brain networks. Each cell displays the coefficient, the corresponding 95% CI, and the FDR-adjusted P value. Asterisks (*) indicate statistically significant differences (*P* < 0.05). The color scale represents correlation coefficients, ranging from negative (blue) to positive (red) correlations. CONT, control network; DMN, default mode network; DAN, dorsal attention network; LIM, limbic network; VAN, ventral attention network; SMN, somatomotor network; SUB, subcortical network; VIS, visual network; MetS, metabolic syndrome; BP, blood pressure.

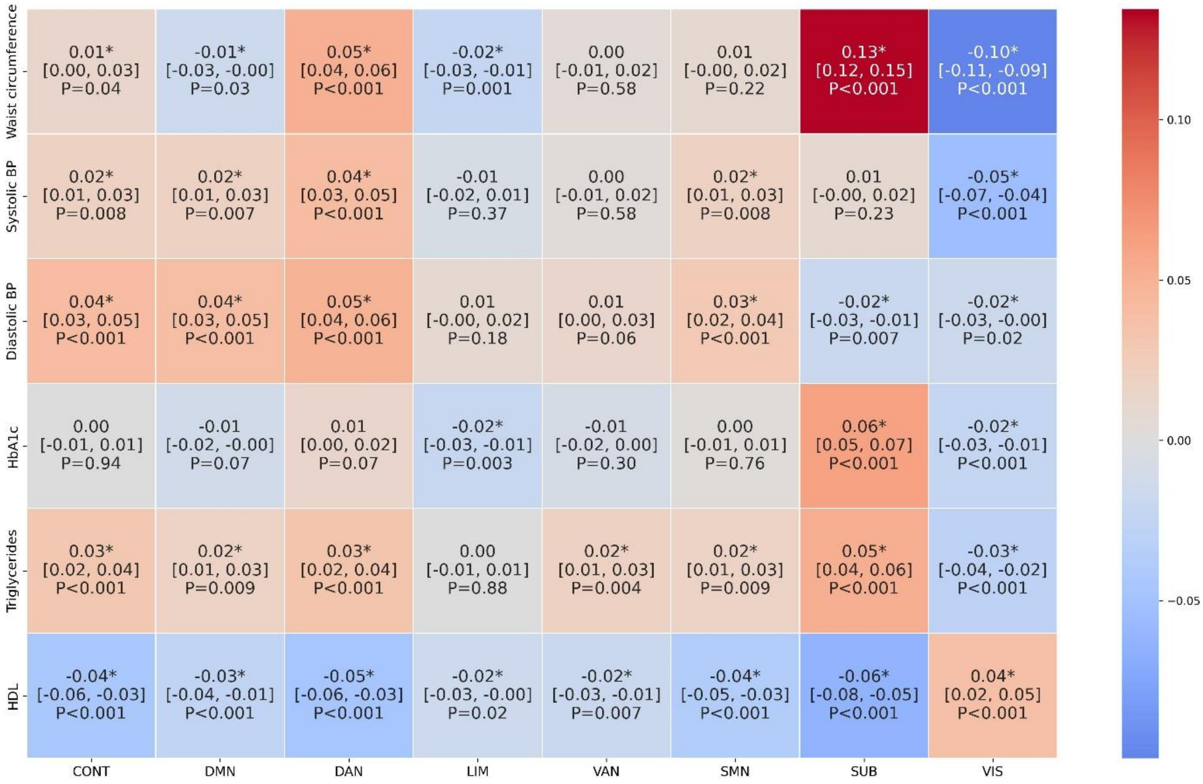

Supplementary Figure S5 Multivariate regression coefficients between selected metabolic indicators and activation energy across major brain networks. Each cell displays the coefficient, the corresponding 95% CI, and the FDR-adjusted P value. Asterisks (*) indicate statistically significant differences (*P* < 0.05). The color scale represents correlation coefficients, ranging from negative (blue) to positive (red) correlations. CONT, control network; DMN, default mode network; DAN, dorsal attention network; LIM, limbic network; VAN, ventral attention network; SMN, somatomotor network; SUB, subcortical network; VIS, visual network; MetS, metabolic syndrome; BP, blood pressure.

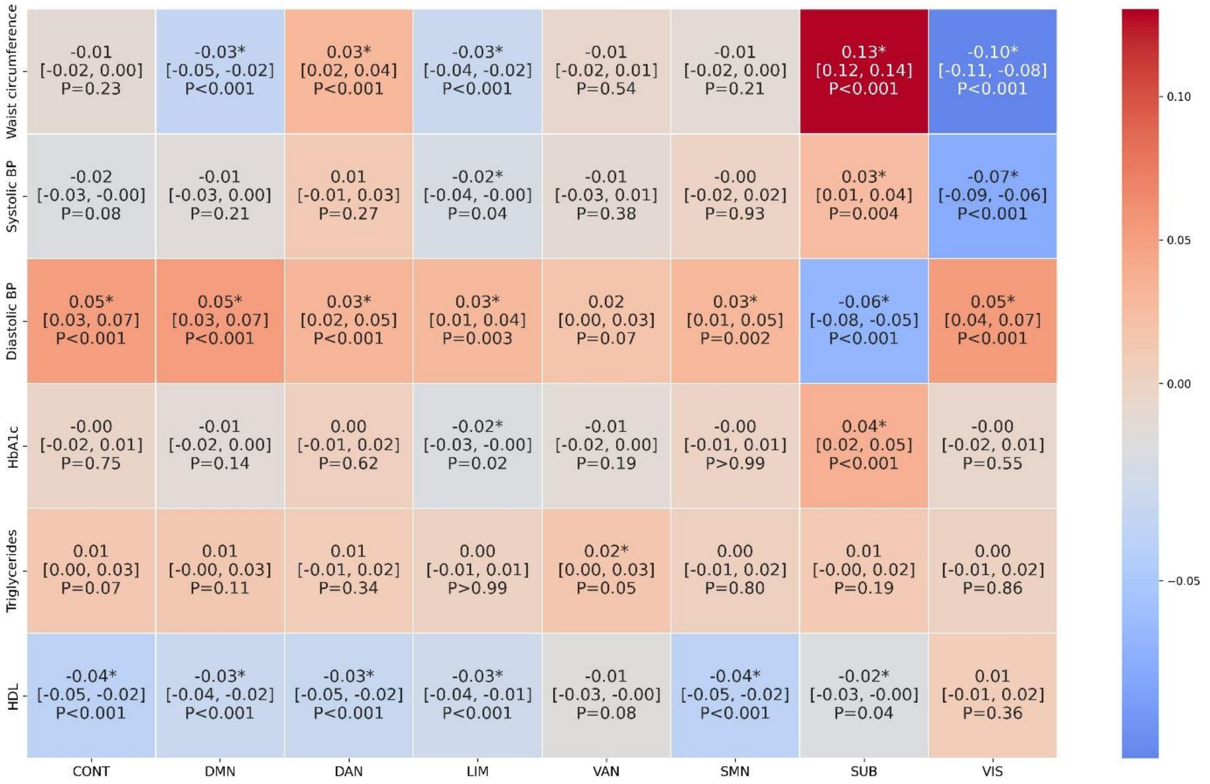

Supplementary Figure S6 Multivariate associations between metabolic features and brain network activation energy via PLSC (age: 40–49 years). CONT, control network; DMN, default mode network; DAN, dorsal attention network; LIM, limbic network; VAN, ventral attention network; SMN, somatomotor network; SUB, subcortical network; VIS, visual network; HDL, high density lipoprotein cholesterol; BP, blood pressure; PLS, partial least squares.

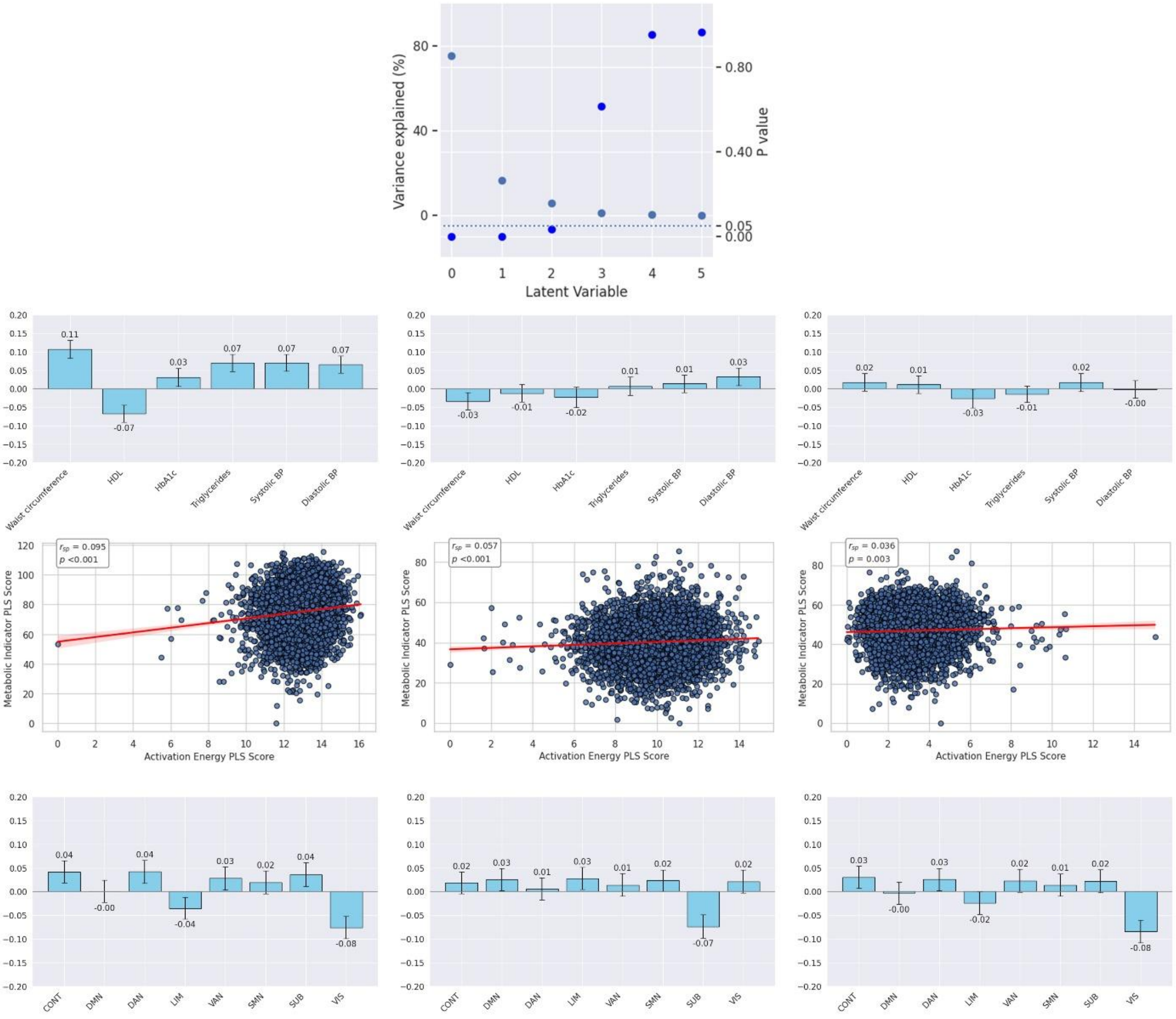

Supplementary Figure S7 Multivariate associations between metabolic features and brain network activation energy via PLSC (age: 50–59 years). CONT, control network; DMN, default mode network; DAN, dorsal attention network; LIM, limbic network; VAN, ventral attention network; SMN, somatomotor network; SUB, subcortical network; VIS, visual network; HDL, high density lipoprotein cholesterol; BP, blood pressure; PLS, partial least squares.

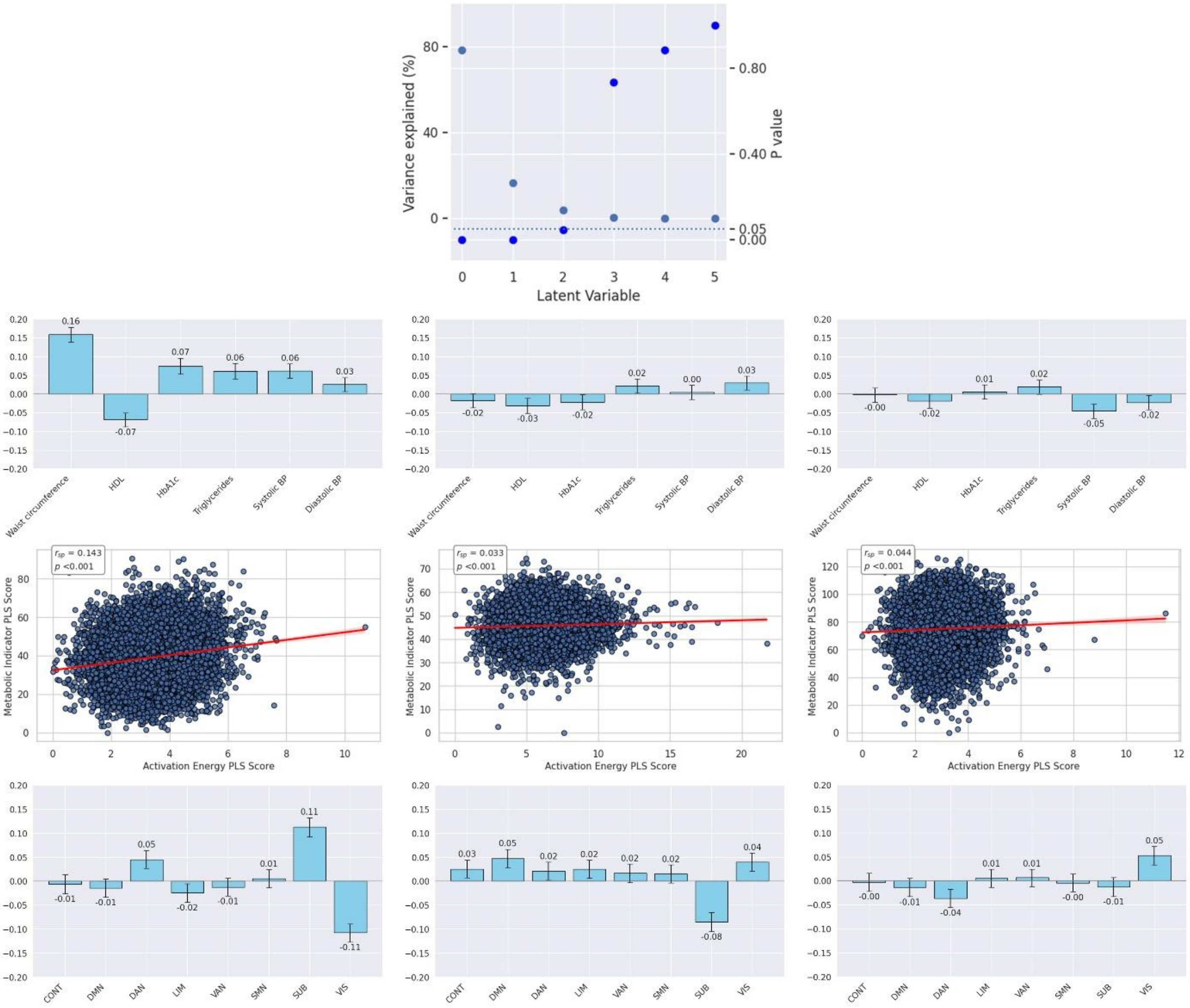

Supplementary Figure S8 Multivariate associations between metabolic features and brain network activation energy via PLSC (age: 60–70 years). CONT, control network; DMN, default mode network; DAN, dorsal attention network; LIM, limbic network; VAN, ventral attention network; SMN, somatomotor network; SUB, subcortical network; VIS, visual network; HDL, high density lipoprotein cholesterol; BP, blood pressure; PLS, partial least squares.

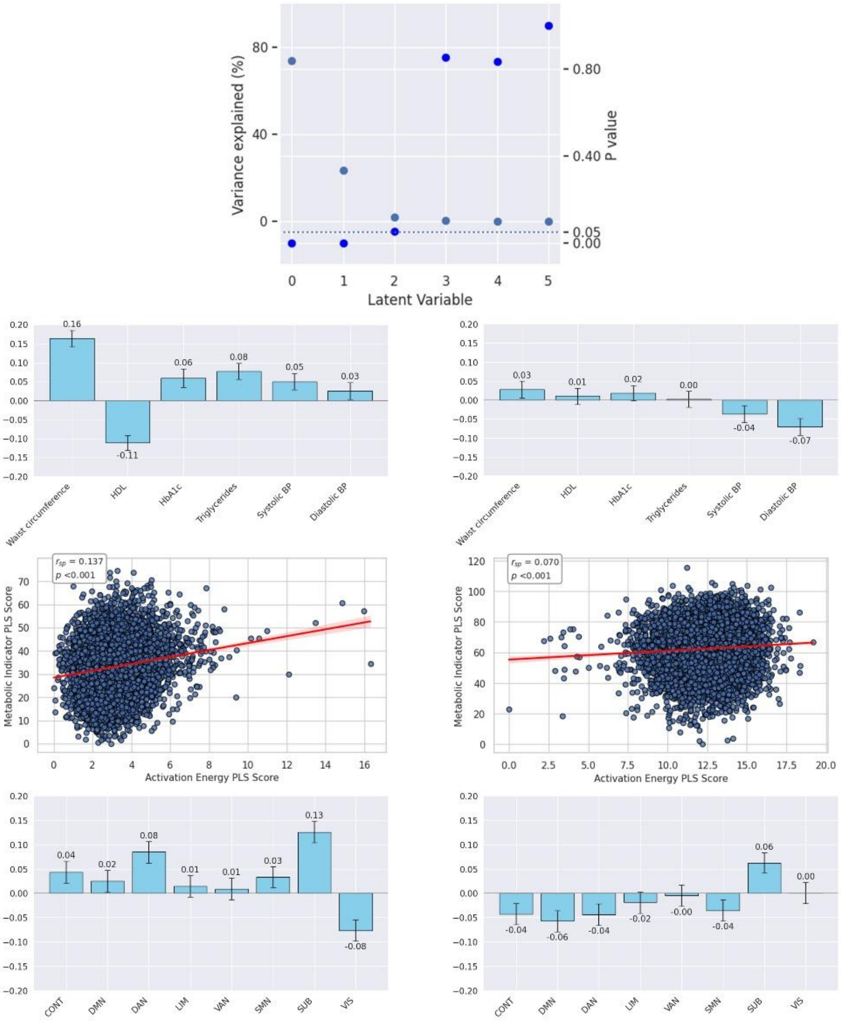

Supplementary Table S1 Exclusion criteria: self-reported neurological and related illnesses.

Self-reported non-cancer illnesses (UK Biobank field 20002)
"dementia", "Alzheimer", "Parkinson", "neurological", "Guillain", "multiple sclerosis", "multiple myeloma", "demyelinating", "brain hemorrhage", "brain abscess", "cerebral", "encephalitis", "epilepsy", "head injury", "meningioma", "meningitis", "motor neuron disease", "spina bifida", "subdural", "subarachnoid"
**Self-reported cancers (UK Biobank field 20001)**
"brain cancer", "meningeal cancer"

Note-Participants were excluded if they self-reported any of the following medical conditions during verbal interviews. Keywords associated with neurological and related systemic illnesses were used to identify relevant conditions in the medical condition field.

Supplementary Table S2 UK Biobank variables and corresponding field numbers.

Variable / Assessment	UK Biobank field number
Waist circumference	48
High-density lipoprotein cholesterol (HDL)	30760
Triglycerides	30870
Systolic blood pressure (SBP)	4080
Diastolic blood pressure (DBP)	4079
Glycated hemoglobin (HbA1c)	30750
Fluid intelligence (FI)	20016
Matrix pattern completion (MPC)	6373
Paired associate learning (PAL)	20197
Symbol digit substitution (SDS)	23324
Trail making test A (TMT-A)	6348
Trail making test B (TMT-B)	6350
Backward digit span (BDS)	4282
Structural connectome	31024

Supplementary Table S3 Selected 123 Neurosynth terms used for cognitive state mapping.

Term list			
action	emotion regulation	judgment	reinforcement learning
adaptation	empathy	knowledge	response inhibition
addiction	encoding	language	response selection
anticipation	episodic memory	language comprehension	retention
anxiety	expectancy	learning	retrieval
arousal	expertise	listening	reward anticipation
association	extinction	localization	rhythm
attention	face recognition	loss	risk
autobiographical memory	facial expression	maintenance	rule
balance	familiarity	manipulation	salience
belief	fear	meaning	search
categorization	fixation	memory	selective attention
cognitive control	focus	memory retrieval	semantic memory
communication	gaze	mental imagery	sentence comprehension
competition	goal	monitoring	skill
concept	hyperactivity	mood	sleep
consciousness	imagery	morphology	social cognition
consolidation	impulsivity	motor control	spatial attention
context	induction	movement	speech perception
coordination	inference	multisensory	speech production
decision	inhibition	naming	strategy
decision making	insight	navigation	strength
detection	integration	object recognition	stress
discrimination	intelligence	pain	sustained attention
distraction	intention	perception	task difficulty
eating	interference	planning	thought
efficiency		priming	uncertainty
effort		psychosis	updating
emotion		reading	utility
		reasoning	valence
		recall	verbal fluency
		recognition	visual attention
		rehearsal	visual perception
			word recognition
			working memory

Supplementary Table S4 Study cohort information.

Description	MetS Score						
	0	1	2	3	4	5	Total
No. (%)	4457 (17.3)	9126 (35.5)	6938 (27.0)	3576 (13.9)	1349 (5.2)	251 (1.0)	25697
**Metabolic indicator, mean ± standard deviation**							
Age, years	51.78 ± 7.18	55.27 ± 7.47	55.76 ± 7.36	55.99 ± 7.25	56.36 ± 7.10	57.12 ± 6.62	54.97 ± 7.48
Waist circumference, cm	78.35 ± 8.71	83.84 ± 9.81	91.43 ± 10.76	97.53 ± 11.31	102.08 ± 10.89	104.68 ± 11.05	88.00 ± 12.45
HbA1c, mmol/mol	33.29 ± 2.94	34.00 ± 3.46	35.15 ± 4.78	36.74 ± 6.06	39.41 ± 8.24	46.02 ± 8.52	34.97 ± 4.98
HDL, mmol/L	1.68 ± 0.35	1.59 ± 0.36	1.40 ± 0.32	1.24 ± 0.29	1.10 ± 0.22	1.03 ± 0.17	1.48 ± 0.38
Systolic blood pressure, mmHg	118.20 ± 8.23	140.03 ± 17.70	144.06 ± 16.74	146.28 ± 15.99	147.50 ± 15.53	149.65 ± 14.91	138.69 ± 18.53
Diastolic blood pressure, mmHg	70.32 ± 6.87	79.08 ± 9.52	81.19 ± 9.66	82.16 ± 9.51	81.99 ± 9.69	81.61 ± 9.27	78.74 ± 10.01
Triglycerides, mmol/L	1.02 ± 0.31	1.25 ± 0.56	1.90 ± 0.95	2.37 ± 1.06	2.73 ± 1.13	2.99 ± 1.17	1.64 ± 0.95
**Gender, No. (%)**							
Male	1370 (30.7)	4356 (47.7)	3805 (54.8)	1973 (55.2)	655 (48.6)	100 (39.8)	12259 (47.7)
Female	3087 (69.3)	4770 (52.3)	3133 (45.2)	1603 (44.8)	694 (51.4)	151 (60.2)	13438 (52.3)
**Smoking, No. (%)**							
Never	2989 (67.1)	5927 (64.9)	4242 (61.1)	2104 (58.8)	747 (55.4)	144 (57.4)	16153 (62.9)
Previous	1314 (29.5)	2901 (31.8)	2418 (34.9)	1328 (37.1)	544 (40.3)	93 (37.1)	8598 (33.5)
Current	143 (3.2)	273 (3.0)	254 (3.7)	129 (3.6)	50 (3.7)	14 (5.6)	863 (3.4)
Missing data	11 (0.2)	25 (0.3)	24 (0.3)	15 (0.4)	8 (0.6)	0	83 (0.3)
**Alcohol, No. (%)**							
Never	246 (5.5)	478 (5.2)	418 (6.0)	289 (8.1)	141 (10.5)	30 (12.0)	1602 (6.2)
Special occasions	375 (8.4)	768 (8.4)	725 (10.4)	441 (12.3)	224 (16.6)	63 (25.1)	2596 (10.1)
1-3 times / month	509 (11.4)	954 (10.5)	791 (11.4)	462 (12.9)	207 (15.3)	46 (18.3)	2969 (11.6)
1-2 times / week	1294 (29.0)	2408 (26.4)	1768 (25.5)	979 (27.4)	364 (27.0)	56 (22.3)	6869 (26.7)
3-4 times / week	1354 (30.4)	2786 (30.5)	1964 (28.3)	875 (24.5)	267 (19.8)	38 (15.1)	7284 (28.3)
Daily or almost daily	675 (15.1)	1731 (19.0)	1270 (18.3)	529 (14.8)	146 (10.8)	18 (7.2)	4369 (17.0)
Missing data	4 (0.1)	1 (0.0)	2 (0.0)	1 (0.0)	0	0	8 (0.0)
**Imaging center, No. (%)**							
Cheadle	2909 (65.3)	5679 (62.2)	4254 (61.3)	2222 (62.1)	871 (64.6)	149 (59.4)	16084 (62.6)
Reading	802 (18.0)	1270 (13.9)	895 (12.9)	399 (11.2)	122 (9.0)	28 (11.2)	3516 (13.7)
Newcastle	738 (16.6)	2163 (23.7)	1783 (25.7)	948 (26.5)	353 (26.2)	74 (29.5)	6059 (23.6)

Bristol	8 (0.2)	14 (0.2)	6 (0.1)	7 (0.2)	3 (0.2)	0	38 (0.1)
**Household income, No. (%)**							
<18000	420 (9.4)	925 (10.1)	763 (11.0)	458 (12.8)	196 (14.5)	40 (15.9)	2802 (10.9)
18000-30999	915 (20.5)	2108 (23.1)	1827 (26.3)	960 (26.8)	422 (31.3)	87 (34.7)	6319 (24.6)
31000-51999	1171 (26.3)	2613 (28.6)	1935 (27.9)	979 (27.4)	338 (25.1)	62 (24.7)	7098 (27.6)
52000-100000	1153 (25.9)	1947 (21.3)	1396 (20.1)	642 (18.0)	208 (15.4)	32 (12.7)	5378 (20.9)
>100000	430 (9.6)	666 (7.3)	408 (5.9)	160 (4.5)	38 (2.8)	7 (2.8)	1709 (6.7)
Missing data	368 (8.3)	867 (9.5)	609 (8.8)	377 (10.5)	147 (10.9)	23 (9.2)	2391 (10.7)
**Education, No. (%)**							
College of University degree	2524 (56.6)	4654 (51.0)	3165 (45.6)	1526 (42.7)	529 (39.2)	99 (39.4)	12497 (48.6)
A levels/AS levels	577 (12.9)	1094 (12.0)	838 (12.1)	449 (12.6)	151 (11.2)	25 (10.0)	3134 (12.2)
O levels/GCSEs	1198 (26.9)	2866 (31.4)	2445 (35.2)	1274 (35.6)	537 (39.8)	102 (40.6)	8422 (32.8)
None of the above	145 (3.3)	482 (5.3)	462 (6.7)	306 (8.6)	129 (9.6)	23 (9.2)	1547 (6.0)
Missing data	13 (0.3)	30 (0.3)	28 (0.4)	21 (0.6)	3 (0.2)	2 (0.8)	97 (0.4)
**Ethnicity, No. (%)**							
White	4320 (96.9)	8889 (97.4)	6717 (96.8)	3445 (96.3)	1309 (97.0)	244 (97.2)	24924 (97.0)
Non-white	125 (2.8)	215 (2.4)	203 (2.9)	121 (3.4)	37 (2.7)	7 (2.8)	708 (2.8)
Missing data	12 (0.3)	22 (0.2)	18 (0.3)	10 (0.3)	3 (0.2)	0	65 (0.3)
**Townsend deprivation index, No. (%)**							
1	864 (19.4)	1965 (21.5)	1407 (20.3)	643 (18.0)	256 (19.0)	37 (14.7)	5172 (20.1)
2	882 (19.8)	1873 (20.5)	1443 (20.8)	682 (19.1)	232 (17.2)	48 (19.1)	5160 (20.1)
3	878 (19.7)	1809 (19.8)	1369 (19.7)	717 (20.1)	269 (19.9)	52 (20.7)	5094 (19.8)
4	897 (20.1)	1773 (19.4)	1408 (20.3)	735 (20.6)	275 (20.4)	56 (22.3)	5144 (20.0)
5	936 (21.0)	1706 (18.7)	1311 (18.9)	799 (22.3)	317 (23.5)	58 (23.1)	5127 (20.0)
**Cognitive test, mean ± standard deviation**							
TMT-A	216.50 ± 59.87	225.47 ± 69.55	228.81 ± 74.18	229.86 ± 74.99	232.19 ± 77.78	237.13 ± 87.97	225.89 ± 70.87
TMT-B	535.38 ± 169.00	570.21 ± 215.75	577.59 ± 213.89	586.78 ± 220.88	588.71 ± 216.09	598.91 ± 189.69	569.72 ± 209.07
FI	6.79 ± 1.96	6.68 ± 1.99	6.58 ± 1.99	6.48 ± 2.02	6.46 ± 2.01	6.28 ± 1.90	6.63 ± 1.99
BDS	6.88 ± 1.05	6.81 ± 1.07	6.76 ± 1.06	6.70 ± 1.08	6.65 ± 1.10	6.56 ± 1.05	6.78 ± 1.07
SDS	19.88 ± 4.19	19.05 ± 4.29	18.77 ± 4.34	18.56 ± 4.31	18.29 ± 4.49	17.87 ± 4.04	19.00 ± 4.32
PAL	7.36 ± 2.05	7.04 ± 2.17	6.83 ± 2.18	6.67 ± 2.23	6.69 ± 2.24	6.37 ± 2.30	6.96 ± 2.18
MPC	8.24 ± 1.69	8.04 ± 1.80	7.96 ± 1.77	7.92 ± 1.86	7.83 ± 1.85	7.64 ± 1.80	8.02 ± 1.79

Note-MetS, metabolic syndrome; HDL, high-density lipoprotein cholesterol; TMT, trail making test; FI, fluid intelligence; BDS, backward digit span; SDS, symbol digit substitution; PAL, paired associate learning; MPT, matrix pattern completion. *$P < 0.05$.

Supplementary Table S5 Model performance and validation metrics for network-specific normative models.

Network	EXPV	MACE	MAPE	MSLL	NLL	R^2	RMSE	SMSE	Rho	*P* value
SUB	0.066	0.019	0.033	-4.314	1.391	0.066	70.274	0.934	0.237	< 0.001*
VIS	0.085	0.022	0.054	-4.737	1.405	0.085	107.672	0.915	0.287	< 0.001*
DAN	0.041	0.015	0.018	-3.814	1.405	0.041	43.791	0.959	0.203	< 0.001*
SMN	0.046	0.019	0.022	-4.013	1.405	0.046	53.267	0.954	0.222	< 0.001*
CONT	0.142	0.013	0.016	-3.855	1.353	0.141	40.979	0.859	0.382	< 0.001*
LIM	0.073	0.017	0.030	-4.317	1.406	0.073	71.198	0.927	0.266	< 0.001*
VAN	0.102	0.015	0.014	-3.697	1.373	0.102	36.511	0.898	0.323	< 0.001*
DMN	0.128	0.018	0.022	-4.066	1.359	0.127	51.328	0.873	0.365	< 0.001*

Note-CONT, control network; DMN, default mode network; DAN, dorsal attention network; LIM, limbic network; VAN, ventral attention network; SMN, somatomotor network; SUB, subcortical network; VIS, visual network; EXPV, explained variance; MACE, mean absolute centile error; MAPE, mean absolute percentage error; NLL, negative loge likelihood; R^2, R-squared; RMSE, root mean square error; SMSE, standardized mean squared error; Rho, Spearman's rank correlation coefficient.

Supplementary Table S6 One-way ANOVA results for the effect of MetS score on activation energy across brain networks.

Network	F-statistic	*P* value
SUB	52.18	< 0.001*
VIS	32.58	< 0.001*
DAN	16.34	< 0.001*
SMN	4.36	< 0.001*
CONT	4.43	< 0.001*
LIM	3.03	0.01*
VAN	2.15	0.06
DMN	2.65	0.02*

Note-CONT, control network; DMN, default mode network; DAN, dorsal attention network; LIM, limbic network; VAN, ventral attention network; SMN, somatomotor network; SUB, subcortical network; VIS, visual network; MetS, metabolic syndrome. *$P < 0.05$.

Supplementary Table S7 Multivariate ANOVA for brain activation energy differences across MetS score groups.

Test statistic	Value	Num DF	Den DF	F-statistic	*P* value
**MetS score 4**					
Wilks' lambda	0.97	32.00	4932.21	1.15	0.25
Pillai's trace	0.03	32.00	5360.00	1.15	0.25
Hotelling-Lawley trace	0.03	32.00	3486.10	1.15	0.25
Roy's greatest root	0.01	8.00	1340.00	2.35	0.02*
**MetS score 3**					
Wilks' lambda	0.97	72.00	21656.13	1.71	< 0.001*
Pillai's trace	0.03	72.00	28528.00	1.70	< 0.001*
Hotelling-Lawley trace	0.03	72.00	14606.23	1.71	< 0.001*
Roy's greatest root	0.01	9.00	3566.00	5.83	< 0.001*
**MetS score 2**					
Wilks' lambda	0.98	72.00	42106.38	2.13	< 0.001*
Pillai's trace	0.02	72.00	55424.00	2.12	< 0.001*
Hotelling-Lawley trace	0.02	72.0000	28427.78	2.13	< 0.001*
Roy's greatest root	0.01	9.0000	6928.00	9.90	< 0.001*
**MetS score 1**					
Wilks' lambda	0.99	32.00	33612.37	2.87	< 0.001*
Pillai's trace	0.01	32.00	36468.00	2.87	< 0.001*
Hotelling-Lawley trace	0.01	32.00	23825.93	2.87	< 0.001*
Roy's greatest root	0.01	8.00	9117.00	6.22	< 0.001*

Note-Num DF, Numerator Degrees of Freedom; Den DF, Denominator Degrees of Freedom. *$P < 0.05$.

Supplementary Table S8 Univariate ANOVA and post-hoc comparison results using Tukey's HSD test for network-level brain activation across MetS score groups.

Network	F-statistic	*P* value	Significant difference
**MetS score 4**			
DAN	1.80	0.13	
VAN	1.72	0.14	
CONT	1.35	0.25	
SUB	1.26	0.28	
VIS	1.24	0.29	
LIM	1.19	0.31	
DMN	0.55	0.70	
SMN	0.47	0.76	
**MetS score 3**			
VIS	2.96	0.002*	BP|HDL|Triglycerides - BP|HDL|Waist circumference<br>BP|HDL|Waist circumference - BP|HbA1c|Triglycerides
SUB	2.70	0.004*	BP|HDL|Triglycerides - BP|Triglycerides|Waist circumference
LIM	1.90	0.04*	BP|HbA1c|Triglycerides - BP|HbA1c|Waist circumference
SMN	1.49	0.15	
DMN	1.03	0.41	
DAN	1.01	0.43	
CONT	0.72	0.70	
VAN	0.29	0.98	
**MetS score 2**			
SUB	6.23	< 0.001*	BP|Triglycerides - BP|Waist circumference<br>BP|Triglycerides - HDL|Waist circumference<br>BP|Triglycerides - Triglycerides|Waist circumference
VIS	3.92	< 0.001*	BP|Triglycerides - BP|Waist circumference<br>BP|Triglycerides - HDL|Waist circumference
DMN	1.88	0.05	
LIM	1.58	0.11	
DAN	1.14	0.33	
SMN	1.08	0.37	
CONT	1.07	0.38	
VAN	0.55	0.84	
**MetS score 1**			
SUB	9.95	< 0.001*	BP - Waist circumference

			HbA1c - Waist circumference
			Triglycerides - Waist circumference
VIS	3.44	0.008*	HbA1c - Waist circumference
DMN	1.78	0.13	
SMN	1.60	0.17	
DAN	1.10	0.36	
CONT	0.80	0.52	
VAN	0.74	0.56	
LIM	0.37	0.83	

Note-CONT, control network; DMN, default mode network; DAN, dorsal attention network; LIM, limbic network; VAN, ventral attention network; SMN, somatomotor network; SUB, subcortical network; VIS, visual network; MetS, metabolic syndrome; BP, blood pressure; HDL, high-density lipoprotein cholesterol. *$P < 0.05$.

Supplementary Table S9 Effect of MetS diagnosis on cognitive test performance.

Cognitive test	Regression coefficient (95% CI)	*P* value
TMT-A	-0.02 (-0.05 – 0.01)	0.35
TMT-B	-0.01 (-0.04 – 0.01)	0.40
FI	-0.02 (-0.05 – 0.01)	0.23
BDS	-0.07 (-0.10 – -0.04)	< 0.001*
SDS	-0.06 (-0.09 – -0.03)	< 0.001*
PAL	-0.09 (-0.11 – -0.06)	< 0.001*
MPC	-0.01 (-0.04 – 0.02)	0.43

Note-TMT, trail making test; FI, fluid intelligence; BDS, backward digit span; SDS, symbol digit substitution; PAL, paired associate learning; MPT, matrix pattern completion; MetS, metabolic syndrome. $^*P < 0.05$.
